# Reconfigurable high-dimensional synthetic photonic lattices

**DOI:** 10.1126/sciadv.adw7198

**Published:** 2025-07-18

**Authors:** Haiqi Huang, Zhuochen Du, Kun Liao, Xiaoyong Hu, Qihuang Gong

**Affiliations:** ^1^ State Key Laboratory for Mesoscopic Physics and Department of Physics, Collaborative Innovation Center of Quantum Matter and Frontiers Science Center for Nano-optoelectronics, Peking University, Beijing 100871, P. R. China.; ^2^ Peking University Yangtze Delta Institute of Optoelectronics, Nantong, Jiangsu 226010, P. R. China.; ^3^ Collaborative Innovation Center of Extreme Optics, Shanxi University, Taiyuan, Shanxi 030006, P. R. China.; ^4^ Hefei National Laboratory, Hefei 230088, P. R. China.

## Abstract

Reconfigurable high-dimensional synthetic photonic lattices offer a promising platform for exploring the dynamic evolution of complex physical systems in high-dimensional spaces. However, the realization of reconfigurable high-dimensional synthetic lattices remains a notable challenge to date. Here, we propose a strategy for realizing dimensionally scalable temporal-domain synthetic photonic lattices, which enables independent control over both the phase and intensity of the signal light at each lattice site and time step within a high-dimensional space. By introducing multiple link rings to realize coupling between site rings, this strategy facilitates the exploration of a wide range of complex physical phenomena. We experimentally validate this proposed strategy in an optical fiber ring system, demonstrating nonreciprocal couplings up to three-dimensional space, topological funnel states, and periodic non-Hermitian temporal modulation to two-dimensional space. Moreover, we theoretically discuss the Weyl surfaces in five-dimensional space. This work provides a platform for exploring high-dimensional physics and paves the way for simulating complex systems, such as quantum many-body models.

## Teaser

A reconfigurable high-dimensional synthetic photonic lattice platform enables the exploration of complex physical phenomena.

## INTRODUCTION

**Fig. 1.**
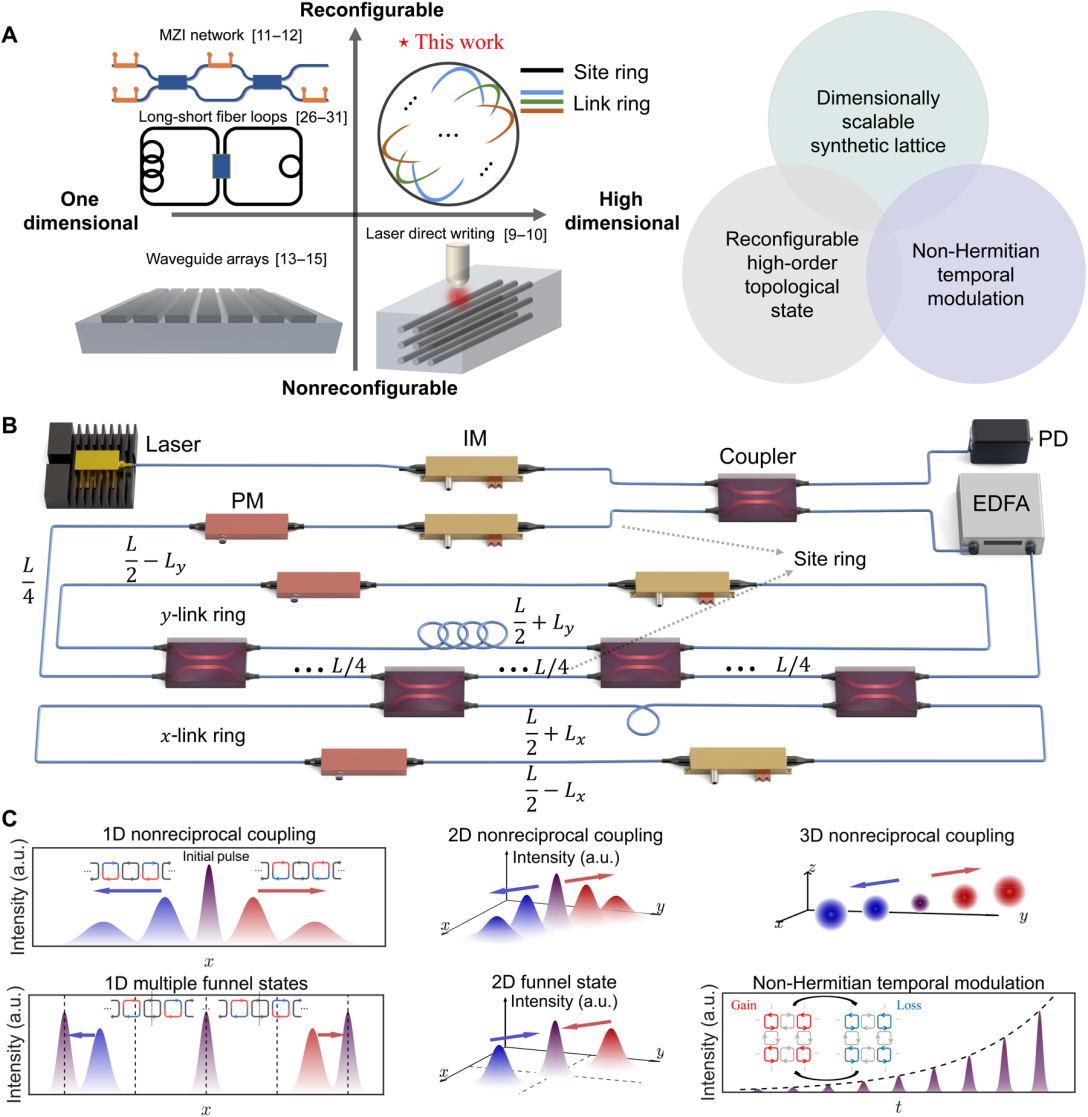
The proposed strategy of reconfigurable high-dimensional synthetic lattice. (**A**) Schematic of simulating state evolution in optical systems. In the upper right, our structure is depicted, consisting of a site ring and several link rings. Each period, the site ring and link rings are coupled twice. The schematic on the right illustrates the implementation achieved in this work: dimensionally extensible synthetic lattices, reconfigurable high-order topological states, and non-Hermitian temporal modulation. (**B**) Schematic diagram of the 2D experiment. The ring at the bottom is the x-link ring, above which lies the site ring, with the y-link ring located inside the site ring. The continuous wave emitted by the laser with a wavelength of 1550 nm is modulated into pulses by an intensity modulator before being input. Each ring contains both an intensity modulator and a phase modulator, as well as an EDFA (depicted only in the site ring due to space constraints). The fiber lengths between adjacent couplers are indicated in the diagram. The intensity modulator is controlled by an arbitrary waveform generator to produce a pulsed signal as the system input. The site ring and the link ring are connected together through 50/50 couplers. The output signal is fed into a PD, which converts the electrical signal to the oscilloscope for display. IM, intensity modulator; PM, phase modulator. (**C**) Schematic diagram of the evolution process for nonreciprocal coupling up to 3D, funnel states up to 2D, and non-Hermitian temporal modulation. a.u., arbitrary units.

The study of the dynamic evolution of high-dimensional complex physical systems is now one of the forefront research areas in the intersection fields of optics and condensed matter. Traditional research platforms, such as cold atom systems (*1*, *2*), trapped ion systems (*3*, *4*), nuclear and electronic spin systems (*5*, *6*), superconducting circuit systems (*7*, *8*), and photonic analog simulators (*9*–*15*), face substantial limitations in terms of configuration flexibility and scalability across dimensions. One possible solution is to use synthetic dimensions (*16*), which are superimposed onto the real space dimensions, enabling the exploration of dynamic evolution of complex physical systems in higher-dimensional spaces. Synthetic dimensions can be realized by leveraging the intrinsic degrees of freedom of photons, such as frequency (*16*–*22*) and orbital angular momentum (OAM) (*23*–*25*), to construct synthetic lattices. The core concept in building a synthetic lattice involves introducing coupling within the intrinsic degrees of freedom. For instance, in the case of frequency synthetic lattice, the discrete resonant frequencies of the optical fiber ring are typically assigned to the lattice sites. By introducing a phase modulated signal with a frequency that is an integer multiple of the fiber ring’s free spectral range, coupling between sites can be achieved (*17*, *18*). Furthermore, by controlling gain and loss through an intensity modulator, a non-Hermitian system can be implemented (*20*, *21*). In addition, the topological charge of optical OAM can be controlled using a liquid crystal or wave plate, facilitating the conversion of the OAM of light. This process is effectively described by an evolution matrix, enabling the realization of a synthetic lattice in the OAM parameter space (*23*, *25*). Nevertheless, the traditional synthetic dimension schemes are typically designed to address steady-state problems, which facilitate the determination of the energy band but poses challenges in analyzing dynamic processes within the lattice. In contrast, synthetic lattices in the temporal degree of freedom are particularly suitable for investigating dynamic processes. The typical synthetic temporal lattice in optical fiber system consists of two coupled optical fiber rings of differing lengths (*26*). When light propagates through the two rings, a time difference is introduced after one complete revolution. After passing through a coupler, the light propagating in the shorter ring effectively equivalent behaves as if it were propagating in the negative direction within the synthetic lattice and vice versa. In optical systems, several approaches are used to study the state evolution process (shown in Fig. 1A), such as laser direct writing waveguide arrays (*9*, *10*), Mach-Zehnder interferometers (MZIs) (*11*, *12*), and on-chip waveguide arrays (*13*–*15*). However, these methods are limited to one-dimensional (1D) configurations or lack reconfigurability. Synthetic temporal lattices offer a valuable research platform for studying evolution processes, particularly in topological photonics (*27*, *28*), non-Hermitian physics (*26*–*29*), disorder phenomena (*30*, *31*), etc. Several studies have realized 2D synthetic lattices and demonstrated corresponding phenomena (*32*–*36*). However, the realization of dimensionally scalable temporal-domain synthetic lattices remains challenging due to the inherent difficulties in extending configuration dimensions and implementing complex coupling schemes such as spin-orbit coupling (SOC).

Here, we propose a strategy to realize a dimensionally scalable temporal-domain synthetic lattices based on an optical fiber ring system, which is well-suited for studying high-dimensional complex physical systems. In an N-dimensional problem, we use one site ring and N link rings to create synthetic lattice. For the 1D case, the site ring and the link ring, having the same length, are coupled by two couplers. For the 2D case, as shown in Fig. 1B, the structure consists of site ring and two link rings. The site ring undergoes four couplings during the coupling process, while each link ring only experiences two. The total lengths of the site ring and the link rings are identical, but the length difference between the two segmented sections of each link ring (separated by the coupler) differs by an order of magnitude. The varying lengths between the two couplers enable the measurement of the dynamic evolution process (see note S8 for more experimental details). This strategy can be extended to higher dimensions. First, we introduce gain and loss in different sections of the link rings to realize nonreciprocal coupling. The resulting unidirectional transmission from nonreciprocal coupling is demonstrated in 1D, 2D, and 3D spaces experimentally. Subsequently, we experimentally implement non-Hermitian domain walls (NH domain walls) using an intensity modulator, enabling the construction of topological funnel states and the observation of their dynamic formation process in both 1D and 2D spaces. Then, time-periodic gain and loss are introduced on the site rings to realize a momentum bandgap, and the dynamic evolution process under time-periodic modulation is experimentally observed in 2D space. Moreover, the site ring and link ring serve different functions in our scheme, unlike in previous works where the rings were treated identically. Our configuration allows for various complex coupling mechanisms, such as SOC, which is challenging to achieve with the setups used in earlier studies. Thus, last, we theoretically propose a method for constructing SOC (detailed in note S5), which enables the formation of Weyl points in 3D space and Weyl surfaces in 5D space. This work provides a valuable approach to studying complex dynamic processes in high-dimensional systems and offering a promising platform for discovering and investigating complex physical phenomena.

## RESULTS

We consider a coupled resonator ring array model, where site rings are interconnected via link rings across different dimensions (*37*). This interaction can be described by a Floquet process. As an example, we consider a 2D system, characterized by the following Hamiltonian

$$H\left(t\right)=\sum_{i,j}\left\{ \begin{array}{cc} \kappa_{x1}\;c_{i,j}^{†}\;a_{i,j} & \mathrm{nT}<t<\left(n+\frac{1}{4}\right)T\\ \kappa_{y1}c_{i,j}^{†}\;b_{i,j} & \left(n+\frac{1}{4}\right)T<t<\left(n+\frac{2}{4}\right)T\\ \kappa_{x2}\;c_{i,j}^{†}\;a_{i-1,j} & \left(n+\frac{2}{4}\right)T<t<\left(n+\frac{3}{4}\right)T\;\\ \kappa_{y2}\;c_{i,j}^{†}\;b_{i,j-1} & \left(n+\frac{3}{4}\right)T<t<\left(n+1\right)T \end{array}\right.+h.c$$ (1)

where $\kappa_{x\left(y\right),1\left(2\right)}$ represents the coupling efficiency, $c_{i,j}^{†}\left(c_{i,j}\right)$ denotes the creation (annihilation) operator for the site ring at position (i,j) , while $a_{i,j}^{†}\left(a_{i,j}\right)$ and $b_{i,j}^{†}\left(b_{i,j}\right)$ correspond to the creation (annihilation) operators for the x-link andy-link rings, respectively. This Hamiltonian describes the coupling between two rings in optics, analogous to the hopping of an electron between atoms in condensed matter physics. In optical fibers, a coupler can facilitate the coupling of two fibers, with the transmission matrix of the coupler representing the time integration of each term in the Hamiltonian.

The core concept of the temporal synthetic lattice based on optical fiber rings is to demonstrate that the dynamics of light in a coupled ring resonator array are equivalent to those in the synthetic lattice. The total length of the site ring is 2L , with the lengths of the upper and lower segments both equal to L . For the *x*-link ring, the upper and lower segments lengths are designed as $L\;/\;2-L_{x}\;$ and $L\;/\;2+L_{x}$ , respectively. Similarly, for other link rings, the lengths of the upper and lower segments are adjusted in the same form. Let T denotes the time taken for light to complete one round trip in the fiber ring. For one of the link rings, such as the x-dimension link ring, the propagation time between the first and second couplers is longer than the corresponding propagation time in the site ring, with the difference denoted as $\;\Delta T_{x}$ . After the second coupling, the time required for the light to complete another round trip and return to the first coupling is shorter than the corresponding propagation time in the site ring, with the time difference denoted as $-\;\Delta T_{x}$ . The same principle applies to link rings representing other dimensions. As an example, in the 2D case, a pulse observed at time $t=\mathrm{nT}+m\Delta T_{x}+l\Delta T_{y}$ corresponds to the wave intensity at time step n and position (m,l) . To avoid finite-size effects, the condition $\Delta T_{x}\ll \Delta T_{y}\ll T$ needs to be satisfied. The lattice numbers are limited by $N_{x}=\Delta T_{y}\;/\;\Delta T_{x}$ and $N_{y}=T\;/\;\Delta T_{y}$ . The experimental setup is detailed in Fig. 1B and note S1. In Fig. 1B, the *x*-link ring is located at the bottom, with the site ring positioned above it and the *y*-link ring situated inside the site ring. The laser, emitting a continuous wave at a wavelength of 1550 nm, is modulated into pulses by an intensity modulator before being introduced into the system. Each ring is equipped with both an intensity modulator and a phase modulator, along with an erbium-doped fiber amplifier (EDFA), which is only shown in the site ring due to space limitations. The fiber lengths between adjacent couplers are indicated in the diagram. The intensity modulator is driven by an arbitrary waveform generator to produce the pulsed signal, which serves as the system input. The site ring and the link ring are interconnected via 50/50 couplers. The resulting output signal is directed to a photodetector (PD), which converts the electrical signal for display on the oscilloscope. This configuration allows for arbitrarily expansion in dimensionality. Experiments have successfully demonstrated the synthesis of lattice configurations from 1D to 3D, along with the realization of nonreciprocal coupling-induced unidirectional propagation, reconfigurable high-order topological states, and non-Hermitian temporal modulation (Fig. 1C).

Here, we use a transfer matrix method to discuss the dynamic in fiber rings. The time integral of the Hamiltonian represents the system’s evolution operator, which corresponds to the transmission matrix in the optical fiber rings. The effect of the coupler on light propagation is described by a transfer matrix, formulated as

$$U_{c}=\left[\begin{array}{cc} t & i\sqrt{1-t^{2}}\\ i\sqrt{1-t^{2}} & t \end{array}\right]$$ (2)

It is assumed that the coupler is lossless. In the 1D case, the configuration in this work is topologically equivalent to those in previous studies (*26*–*31*). However, a key distinction lies in the roles of the two rings in our configuration: One serves as a site ring, while the other is a link ring, with each playing different roles. The link ring solely provides coupling for the site ring, whereas in previous works, the long and short rings were treated equivalently in their roles. The link ring serves as a medium for coupling between the site rings. Our primary focus is on the dynamic evolution within the site rings. Unlike previous works, where the long and short rings were treated as having equivalent roles, we highlight their distinct functions. This distinction becomes more pronounced in higher dimensions. In the 2D case, the site ring couples with two link rings twice, resulting in a total of four couplings, while in the 3D case, it couples with three link rings twice, yielding a total of six couplings, the deviations are detailed in note S1. In this configuration, the coupling coefficients of the synthetic lattice can be tuned by adjusting the splitting ratio of the couplers. The introduction of phase modulators in both the site ring and the link rings allows for control of the transmission phase, which is effectively equivalent to the on-site potential of the lattice sites. Furthermore, the incorporation of intensity modulators enables the realization of non-Hermitian processes. The modulator we use has a maximum modulation speed of up to 10 GHz, which is faster than the shortest time difference in the link ring, allowing for independent control of modulation at each site. In addition, the arbitrary waveform generator we use enables the customization of waveforms, ensuring that the modulation signals are independently controllable in each cycle of the optical ring. Such a characteristic is realized in high-dimensional synthetic lattices, providing the potential for studying the evolution of systems in high-dimensional spaces.

### Verification of state dynamical evolution in 1D space

**Fig. 2.**
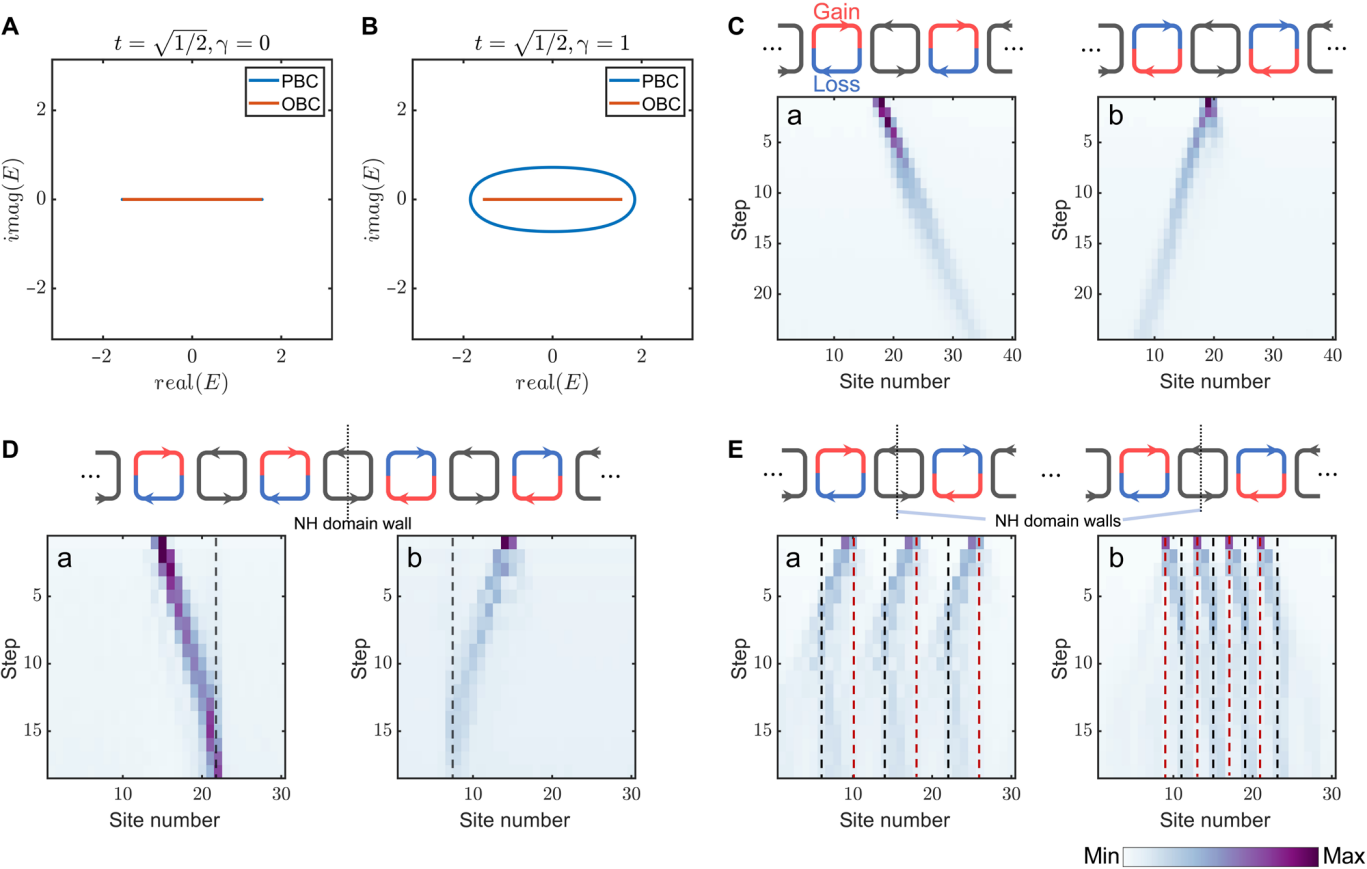
Results of nonreciprocal coupling and funnel states in 1D space. (**A**) Quasi-energy band for reciprocal coupling case with $t=\sqrt{1/2}$ . The results for PBC and OBC nearly coincide. (**B**) Quasi-energy band for nonreciprocal coupling case with $t=\sqrt{1/2}$ and γ=1 . For PBC, the band forms a ring in the complex plane, while for OBC, it appears as a line along the real axis. (**C**) The evolution results of nonreciprocal coupling in the (a) positive and (b) negative directions of the x axis. (**D**) The evolution results of topological funnel state with the input pulse at (a) negative and (b) positive sides of NH domain wall. (**E**) The evolution results of multiple topological funnel states with the input pulses at (a) negative and (b) positive sides of NH domain wall. The black dashed lines represent NH domain walls that can give rise to funnel states, while the red dashed lines correspond to NH domain walls where funnel states do not exist.

To validate the proposed scheme, we implement an experiment in 1D synthetic lattice. We use two 50/50 couplers ( $t=\sqrt{1\;/\;2}$ ) to couple the site ring with the link rings and place a 50/50 coupler on the site ring to inject pulse and detect the output pulses of the site ring after each step of evolution. The output light causes 50% loss in the site ring, which is compensated for by an EDFA placed on the site ring. Initially, the unidirectional propagation effect induced by nonreciprocal coupling is demonstrated. The schematic diagrams are shown in the top of Fig. 2C, and in the lower half of the link ring, an intensity modulator is used to introduce loss, while in the upper half, an EDFA is used to provide optical gain. Using Floquet theory, the complex band structure of the system is calculated for both the reciprocal and nonreciprocal cases. Figure 2A presents the quasi-energy band spectra under periodic boundary condition (PBC) and open boundary condition (OBC) for the reciprocal case. Gain and loss factors of $e^{\pm \gamma }$ are applied to the upper and lower parts of the link ring. The complex band structure was calculated for γ=1 as shown in Fig. 2B. In this case, the quasi-energy bands under PBCs form a ring in the complex band structure, while the complex band structure under OBCs remains purely real, causing the bulk-edge correspondence to break down (*38*). The reason is that in the nonreciprocal coupling case, the translation of the wave function causes the amplitude changed, breaking translation invariance. By defining the generalized Brillouin zone (detailed in note S3), the bulk-edge correspondence can be reconstructed (*39*). In this setup, the light propagating in the negative x direction undergoes attenuation, whereas the light propagating in the positive x direction is amplified. As a result, the pulse exhibits a tendency to propagate in the positive direction which is shown in Fig. 2C (a). Conversely, as depicted in Fig. 2C (b), by applying loss in the upper half of the link ring and gain in the lower half, nonreciprocal coupling in the positive x direction can be achieved, thereby inducing the pulse to propagate in the positive direction. The quasi-energy band structure can be derived from the intensity distribution during the evolutionary process, as detailed in note S7. Both theoretical simulation results and experimental data are presented in fig. S8. In the system, a domain wall is introduced, with nonreciprocal coupling from left to right on the left side and nonreciprocal coupling from right to left on the right side. In this case, the system will exhibit funnel states, where, regardless of the incident light position, the light will always propagate toward the domain wall and remain vicinity of the domain wall. The experimental results for the incident pulse located in the negative direction of the NH domain wall are shown in Fig. 2D (a). The light initially propagates unidirectionally toward the position of the NH domain wall and subsequently becomes localized near the domain wall. The process of incident light on the right side of the NH domain wall is shown in Fig. 2D (b). For Fig. 2D, the initial state is fixed at position 15, while the domain wall is placed at positions 22 and 8, respectively. Here, the nonreciprocal transmission of light in the positive x direction is stronger than in the negative x direction, a phenomenon also observed in the simulations. A similar effect occurs even in reciprocal coupling, which can be attributed to the coupling process where the site ring initially couples with the link ring in the positive direction. In addition, multiple domain walls can be introduced into the system, where the incident light in different regions will localize at different domain walls, thereby forming multiple funnel states. Figure 2E (a) illustrates the situation of six NH domain walls, three of which lead to funnel states. For the three domain walls, the incident pulses are positioned in the positive direction of the corresponding walls and become localized near the domain walls after propagation. Figure 2E (b) shows the results for four funnel states, where the incident pulses are positioned in the negative direction of the corresponding domain walls. The theoretical results corresponding to the above experimental data are shown in fig. S4. These results demonstrate the ability of the proposed scheme to study non-Hermitian topological states in 1D system, and its reconfigurable characteristics enrich the range of physical phenomena that can be realized.

### Nonreciprocal coupling and topological funnel states in 2D space

**Fig. 3.**
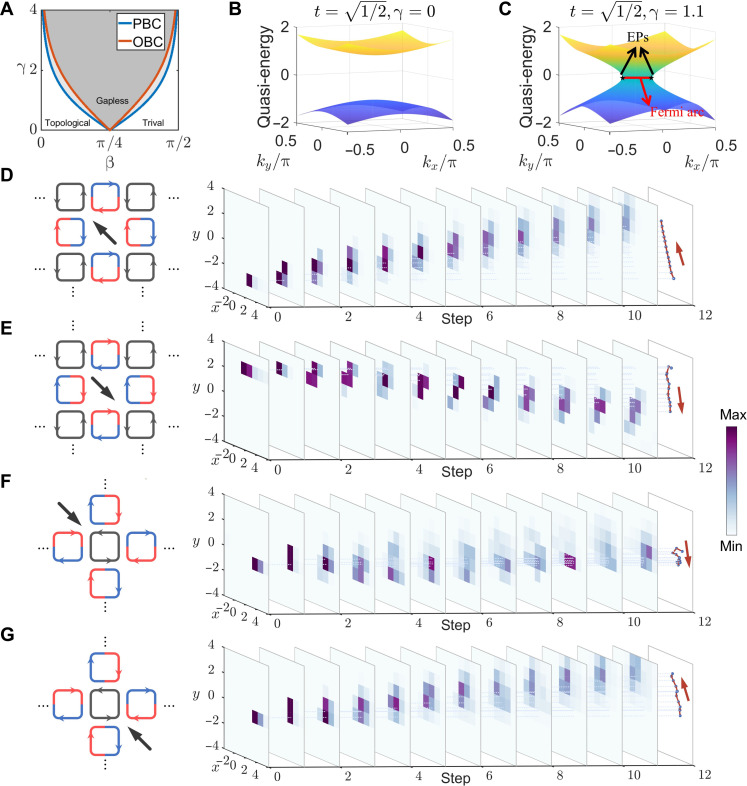
Results of nonreciprocal coupling and funnel state in 2D space. (**A**) The topological phase diagram for 2D nonreciprocal coupling, where the blue line represents the boundary for the PBC case, and the orange line represents the boundary for the OBC case. (**B**) 2D quasi-energy band for reciprocal coupling. (**C**) 2D quasi-energy band for nonreciprocal coupling. In this case, the system is in a gapless phase, with the two black pentagrams representing exceptional points, and the red line indicating the Fermi arc. EPs, exceptional points. (**D**) Schematic of nonreciprocal coupling toward the upper left direction and its experimental results. (**E**) Schematic of nonreciprocal coupling toward the lower right direction and its experimental results. (**F**) Schematic diagram of the funnel state with the input pulse at the upper left direction of the funnel. The right figure is the experiment result. (**G**) Schematic diagram of the funnel state with the input pulse at the lower right direction of the funnel. The right figure is the experiment result.

This approach can be directly extended to 2D, with the key consideration being to maintain equal total lengths for the site ring and both link rings. In Hermitian case, the Floquet system exhibits trivial phase and topological phase with the topological phase transition critical point at $\;t=\sqrt{2}-1$ . In non-Hermitian case, we calculated the topological phase diagram of the 2D system using the coupling coefficient and the gain-loss parameters as variables, as shown in Fig. 3A. Introducing $\text{tan}\beta =\frac{2t}{1-t^{2}}$ as an auxiliary variable can simplify the description of the topological phase diagram (*40*). Because of the failure of the bulk-edge correspondence, the PBC and the OBC exhibit different topological phase boundaries (*38*). The topological phase boundaries calculated in the two cases are

$$\begin{array}{c} \;\text{sin}\left(2\beta \right)\text{cosh}\left(\gamma \right)=1\\ 1-\text{sin}\left(\beta \right)\text{cos}\left(\beta \right)\left(e^{\gamma }+1\right)=\pm \text{cos}\left(\beta \right)\mp \text{sin}\left(\beta \right) \end{array}\;\begin{array}{c} \text{PBC}\\ \text{OBC} \end{array}$$ (3)

where γ is the gain-loss factor defined above. The deviation is detailed in note S4. Previous work shows that applying gain-loss on the site ring can induce a topological phase transition (*40*), but the situation is different from applying gain-loss on the link ring. In this case, the system exhibits three topological phases: trivial, topological, and gapless phases. Regardless of the system’s state in the Hermitian case, after applying sufficiently large gain-loss, it will always enter the gapless phase. The evolution of the bulk energy bands is shown in Fig. 3 (B and C). The quasi-energy bands are plotted relative to π . When $t=\sqrt{1\;/\;2}$ , the system has a trivial bandgap. Applying gain-loss causes the line gap to close. At the zero-energy level in the quasi-energy spectrum, a Fermi arc appears, connecting the two exceptional points. By controlling the gain-loss distribution of the x and y link rings, independently tunable nonreciprocal coupling can be achieved in arbitrary directions. In our experiment, we demonstrate the dynamical evolution of waves during nonreciprocal coupling in the upper left and lower right directions. The results are shown in Fig. 3 (D and E). The final plot of the experiment shows a clear unidirectional propagation of the wave packet’s average position over time. The theoretical results are presented in note S6. The experimental observations qualitatively agree with the theoretical calculations, but there are some differences in the numerical values. These differences may be attributed to the thermal instability of the EDFA and temperature drift of the intensity modulator. Furthermore, we introduce nonreciprocal couplings in four directions within a 2D lattice. In the *x* direction, a domain wall exists, with nonreciprocal coupling directed from the negative side of the wall toward the positive direction and vice versa on the other side. The same applies to the y direction. The mathematical form of this modulation signal is detailed in note S8. Taking the intersection of the two domain walls as the funnel, the coordinate plane is divided into four quadrants, with the direction of nonreciprocal coupling in each quadrant pointing toward the funnel. As a result, incident light tends to propagate toward the funnel when transmitted in different quadrants. To demonstrate such a nonreciprocal coupling process, it is necessary to precisely control the modulation signal on the intensity modulator to achieve a periodic variation of gain and loss over time on the link ring. This temporal variation maps onto the synthetic lattice, creating different gain-loss conditions at different positions and thereby realizing domain walls. With the incident light positions at the lower right and upper left of the funnel, the dynamical process in which light transitions from a bulk state to a funnel state is demonstrated. The experimental results are shown in Fig. 3 (F and G). These two figures illustrate the cases where the funnel state is positioned to the upper left and lower right of the initial state, respectively. After propagating for several periods, the light becomes localized around the vicinity of the funnel state. In the plot of the wave packet’s average position, the incident pulse located at the lower right of the funnel couples toward the upper left and reaches the funnel in the last two steps. During this process, the average position shows little change, and the intensity distribution changes negligibly, which supports this observation. Another phenomenon that can further confirm this is that the initial state in Fig. 3G ( y=−1 ) has a noticeably larger *y* value compared to Fig. 3E ( y=−3 ), yet by the final step, the *y* values of both states converge toward each other. This also demonstrates that in the presence of a funnel state, the state begins to localize as it approaches the funnel state. This behavior fundamentally differs from the previous unidirectional propagation, despite the evolution being quite similar in the earlier steps. The theoretical calculation results for the 2D evolutionary process are shown in fig. S5. The experimental results fit well with the theoretical predictions, demonstrating the reliability of the proposed scheme after dimensional extension. Increasing the gain-loss ratio can further enhance the localization of the funnel state. To better investigate phenomena that only arise in higher dimensions, we explore the physics of the geometry-dependent skin effect. The theoretical results are provided in detail in note S10. To experimentally demonstrate phenomena that exist only in higher dimensions, we also present the non-Hermitian router in note S9, which enables reconfigurable path control through the distribution of gain and loss.

### Exploration of higher-dimensional physics

**Fig. 4.**
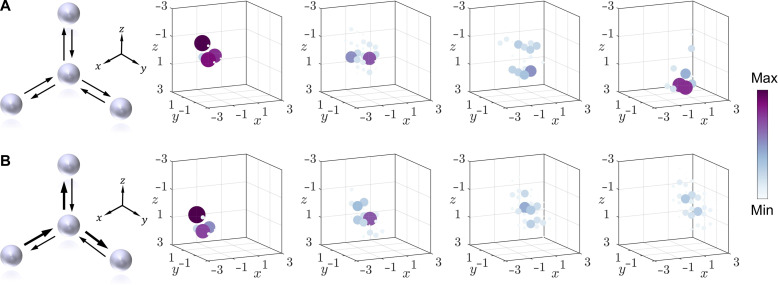
Results of nonreciprocal coupling in 3D space. (**A**) Schematic of 3D reciprocal coupling and experimental results at time steps 1, 3, 5, and 7. (**B**) Schematic of 3D nonreciprocal coupling and experimental results at time steps 1, 3, 5, and 7.

To further demonstrate the scalability of this strategy, we extend the synthetic lattice to 3D and perform experiments. Specifically, the site ring first couples with the link rings in the positive x,y, and z directions and then couples with the link rings in the negativex,y, and z directions. This process directly maps onto the arrangement of optical fiber ring in the experiment. In the experiment, the evolution of a wave packet at T=  1, 3, 5, and 7 in a Hermitian lattice is first demonstrated, as shown in Fig. 4A. Here, the size and color of the balls represent the wave packet intensity distribution at the corresponding positions. Then, following the procedures used in lower dimensions, nonreciprocal coupling is achieved by applying gain and loss to different parts of the link rings. Here, the experimental results at T=  1, 3, 5, and 7 of nonreciprocal coupling in the positive directions of the three coordinate axes are shown in Fig. 4B. The spheres in Fig. 4 (A and B) represent the site rings, and the arrows represent the equivalent coupling of the link rings. In Fig. 4A, the arrows are of the same size, indicating a reciprocal coupling situation. In Fig. 4B, the arrows are of different sizes, indicating a nonreciprocal coupling situation, with larger arrows representing larger equivalent coupling coefficients. Because of the limitation of figure’s size, T=  2, 4, and 6 are detailed in note S9 and fig. S9. The experimental results show unidirectional transmission properties under nonreciprocal coupling, which is consistent with the theoretical expectations. The theoretical results are shown in note S6 and fig. S6.

**Fig. 5.**
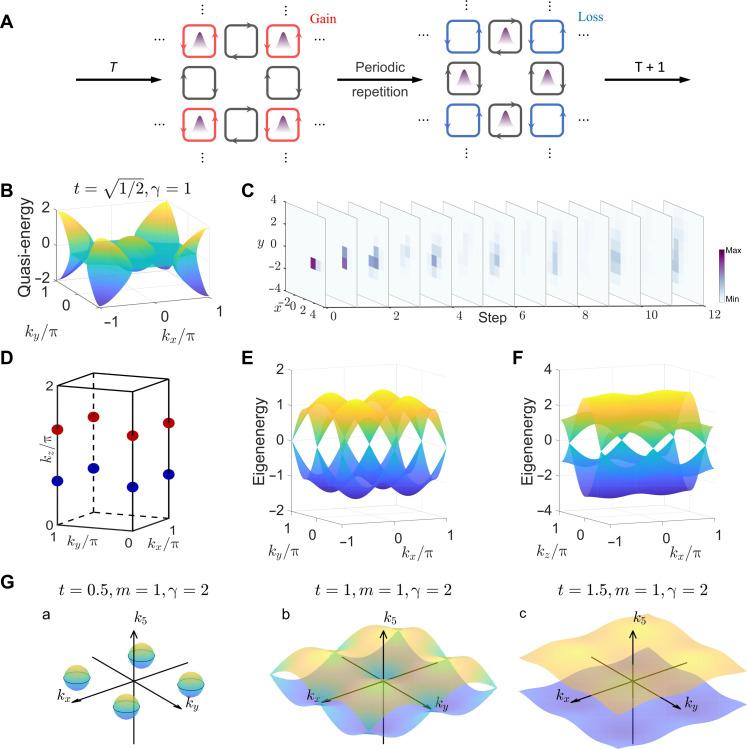
Results of non-Hermitian time modulation and high-dimensional physics. (**A**) Schematic for period non-Hermitian modulation. The site ring exhibits gain in one time step and loss in the subsequent time step, with this process repeating periodically. (**B**) Quasi-energy band under non-Hermitian time modulation. A ring-like momentum bandgap has emerged. (**C**) Experimental result for the non-Hermitian time modulation. (**D**) Position of Weyl points. (**E** and **F**) 3D energy band for $k_{z}=\text{acos}\left(-0.4\right)$ and $k_{y}=0$ . The dispersion relation near Weyl points is linear. (**G**) Schematic of Weyl surfaces for t=0.5,1,and1.5.

In the above cases, the period of the modulation signal is equal to the period of light propagation around the ring. However, the signal can be modulated individually within each period through a specific design to realize complex phenomena. The modulation applied to the light can also be changed in each cycle, meaning that the synthetic lattice dynamically evolves. Consider a dynamically varying lattice with a doubled period, where the site ring acquires a gain after completing its coupling with both link rings. In the subsequent cycle, the site ring loses a portion of the light. This process repeats cyclically, as shown in Fig. 5A. For such a system, because of its inherent spatial translation symmetry, the quasi-energy band structure can be calculated using Floquet theory. It is worth mentioning that the evolution matrix corresponding to two cycles of light propagation is required. When the gain-loss factor reaches a certain threshold, the quasi-energy spectrum opens a ring-like momentum bandgap (*41*–*43*), which is shown in Fig. 5B. The quasi-energy plotted here is relative to π . Light with such quasi-momentum within the lattice cannot propagate and will instead experience exponential increase due to parity-time (PT) symmetry breaking. This phenomenon can be explained by the following process: when the site ring is in the gain condition, the light is mainly confined within the site ring, leading to an increase in the total energy of the system. In the next cycle, the light is mainly confined within the link rings, and the gain of the site ring has a minimal effect on the reduction of the system’s total energy, this process is shown in Fig. 5A. As a result, in these two cycles, the light continuously absorbs energy from the external environment, causing the total energy to diverge exponentially. Because of the symmetry of the system, another state that is related to this one by a PT transformation undergoes the opposite process. As a result, it will experience exponential decay and quickly become unobservable. In the experiment, we realize a time-modulated lattice and observe the phenomenon of forbidden propagation within the momentum bandgap. During the evolution process, the state with the largest gain corresponds to the state with the largest imaginary part in the Floquet eigenstates (the profile of the eigenstate with the largest imaginary component is detailed in note S6). This state exhibits a period doubling behavior during propagation, which was also observed experimentally. To prevent excessive amplification of noise that could interfere with the signal observation, we applied a certain amount of gain-loss translation to the system, shifting the system toward the loss side to ensure an optimal signal-to-noise ratio. The experiment result is shown in Fig. 5C. It can be observed that, after the initial state begins to evolve, the eigenstate with the largest imaginary component dominates. During the periodic gain cycle of the site ring, the state is distributed within the site ring and vice versa. The experimental results fit well with theoretical prediction as shown in note S6.

Some special physical phenomena do not exist in lower-dimensional spaces but emerge in higher-dimensional spaces. A typical example is the appearance of Weyl points and Weyl surfaces. We theoretically propose a scheme to realize SOC in this system. We calculate the Weyl points in 3D space and the emergence of Weyl surfaces in 5D space. Considering that a single-mode optical fiber can support light propagation in polarization directions, the use of a polarization modulator on the optical fiber of the connection ring allows for control over the polarization direction. When the phase difference between the link ring and the site ring is π , and t approaches 1, it can be demonstrated that the system’s dynamic behavior can be described using the Hamiltonian that only considers the site ring (detailed in note S5). The coupling coefficients and phase in the Hamiltonian can be controlled by adjusting the value of t , and the SOC can be manipulated via the polarization controller. The momentum space’s Hamiltonian (*2*) can be written as

$$H\left(k\right)=t_{1}\left(\text{sin}k_{x}\sigma_{x}+\text{sin}k_{y}\sigma_{y}\right)+\left(2t_{3}\text{cos}k_{z}+m\right)\sigma_{z}$$ (4)

where $t_{1}$ represents the strength of SOC, where $t_{3}$ denotes the coupling strength for interlayer interactions, and m is the on-site potential. The deviation is detailed in note S5.

The energy band is given by

$$E_{k}=\pm \sqrt{t_{1}^{2}\left({\text{sin}}^{2}k_{x}+{\text{sin}}^{2}k_{x}\right)+{\left(2t_{3}\text{cos}k_{z}+m\right)}^{2}}$$ (5)

The bandgap closing at $\begin{array}{c} k_{x}=k_{y}=n\pi ,n\in \mathbb{Z},\;\text{cos}k_{z}=-m/2t_{3}\;\left(m<2t_{3}\right). \end{array}$ The system exhibits a pair of Weyl points within the first Brillouin zone. Here, we choose $m=0.8\;\text{and}\;t_{3}=1$ . The positions of the Weyl points are shown in Fig. 5D. Figure 5E shows the energy band with $k_{z}=0$ , and Fig. 5F shows the energy band with $k_{y}=0$. It can be observed that the degeneracy points appear in pairs, and in their vicinity, the dispersion relation is linear in all 3D which confirms the formation of Weyl points.

Aside from controlling the linear polarization to achieve SOC, circularly polarized light can also be used as a basis. In the site ring, a polarization beam splitter is used to separate the linear polarization, with phase modulation applied to the split beams, which also enables the implementation of the spin-flip term, thereby inducing SOC (detailed in note S5).

To realize a Weyl surface, a system with four energy bands needs to be constructed in 5D space. Here, we present a scheme based on a tetramer to achieve the Weyl surface. Here, a four-band system is constructed using the OAM degrees of freedom of multimode fibers. The four modes can be selected as $\begin{array}{c} |\;l=1,x\;\rangle ,|\;l=-1,x\rangle ,|l=1,y\;\rangle ,|\;l=-1,y\;\rangle \end{array}$ , where l denotes the topological charge of OAM and x and y represent the polarization directions.

The Hamiltonian (*44*) in momentum space is

$$\begin{array}{c} H\left(k\right)=t_{1}\left(\text{sin}k_{x}\Gamma_{10}+\text{sin}k_{y}\Gamma_{20}+\text{sin}k_{z}\Gamma_{31}+\text{sin}k_{4}\Gamma_{32}\right)\\ +\left(m+2t_{5}\text{cos}k_{5}\right)\Gamma_{30}+\frac{1}{2}i\gamma \left[\Gamma_{31},\Gamma_{32}\right] \end{array}$$ (6)

where $\Gamma_{\mathrm{ij}}=\sigma_{i}⨂\sigma_{j}\;\text{and}\;\sigma_{1,2,3}=\sigma_{x,y,z}$ are Pauli matrices, and $\sigma_{0}$ is the two-order identity matrix. The method of realizing such a Hamiltonian using the OAM degree of freedom is detailed in note S5. The energy bands are

$$E\left(k\right)=\pm \sqrt{\xi_{31}^{2}+\xi_{32}^{2}+{\left(\gamma \pm \sqrt{\xi_{10}^{2}+\xi_{20}^{2}+\xi_{30}^{2}}\right)}^{2}}$$ (7)

denotes the coefficients that multiply the $\Gamma_{\mathrm{ij}}$ terms in the Hamiltonian. The second-lowest and third-lowest energy bands will form Weyl surfaces

$$\xi_{31}=\xi_{32}=0,\xi_{10}^{2}+\xi_{20}^{2}+\xi_{30}^{2}=\gamma^{2}$$ (8)

This requires $k_{z}=k_{4}=0.$ In the 3D subspace formed by $k_{x}$ , $k_{y}$ , and $k_{5}$, the solutions to the above equation form a 3D surface. The evolution of the Weyl surfaces with varying t is shown in Fig. 5G. Given m=1 and t=2 , as t changes from 0.5 to 1.5, the closed Weyl surfaces will reconnect and then open. A key advantage of the temporal synthetic lattice is its ability to directly observe the evolution. We have theoretically realized a 4D artificial gauge field and showed chiral transport of edge state in the context of the 4D quantum Hall effect, as shown in note S11. The above results demonstrate our system’s ability to realize synthetic lattices in higher-dimensional spaces and exhibit complex physical phenomena, providing a platform for the study of high-dimensional physics.

## DISCUSSION

We have realized the reconfigurable high-dimensional synthetic lattice, demonstrating various physical phenomena, including nonreciprocal coupling-induced unidirectional propagation up to 3D space, topological funnel states up to 2D space, and non-Hermitian temporal modulation. Our work provides a research platform for revealing complex physical laws in high-dimensional spaces. The proposed configuration can be further extended to higher-dimensional spaces, enabling the demonstration of physical models and corresponding phenomena that do not exist in reality. We theoretically discuss the Weyl points in 3D space and Weyl surfaces in 5D space. To further increase the dimensionality of the synthetic lattice, longer optical fibers and shorter pulses are required. In addition, this necessitates the use of signal generators, electro-optic modulators, and PDs with higher operating frequencies. Furthermore, we will consider introducing nonlinear interactions and many-body interactions into the system. The inclusion of these interactions will greatly enrich the range of physical models that can be studied within our framework. Our system provides an even more powerful platform for simulating complex physical processes and investigating their underlying principles.

## MATERIALS AND METHODS

### Experimental setup

The laser used in the experiment is Santec-TSL-510, and an intensity modulator Thorlabs LN82S-FC is used to generate the input pulses. The pulse signal is then amplified by a Thorlabs EDFA-100S and passed through a filter to reduce spontaneous emission noise. A polarization modulator is placed on the optical fiber, followed by a polarizer, ensuring that the propagating signal is polarized along the slow axis. The input pulse is coupled into the site ring through a polarization-maintaining 50:50 coupler. It then enters the Thorlabs EDFA-100P for signal amplification, compensating for the losses caused by the output from the coupler. The amplified light passes through a filter to remove spontaneous emission noise, which is also applied to the link ring. The coupling process then begins, where, to ensure effective coupling, polarization-maintaining fibers and polarization-maintaining couplers are used throughout. Because of the difficulty in fabricating ultra-long polarization-maintaining fibers, a common length is removed from all the rings between the first and second couplings, which does not affect the dynamics of the process. Further details are listed in note S8.

### Data processing

The data are read and saved from the oscilloscope, where the independent variable corresponds to time, and the dependent variable is the voltage signal obtained from the PD. The data processing begins by determining the total time corresponding to the entire optical fiber ring, which corresponds to the number of sampling points for the independent variable. When all the link rings and site ring are disconnected, a series of approximately equally spaced peaks are obtained, which correspond to the pulses circulating within the site ring. The position of each pulse is determined by identifying the local maxima, and a linear fit is then performed to determine the circulating time. Next, the time difference corresponding to each dimensional link ring needs to be determined. For example, for the x-dimension link ring, the system is configured such that only the x-dimension link ring is coupled with the site ring. After a few steps of evolution, a series of equally spaced peaks is obtained. By performing a linear fit, the time difference for the x-dimension link ring, denoted as $\Delta T_{x}$ , can be determined. The time differences for the other dimensions can be determined using the same method. During the processing, it is necessary to adjust the starting point position to control the location of the incident pulse within the coordinate system. The pseudocode for the processing procedure is detailed in note S2 and fig. S3.

## Supplementary Materials

### This PDF file includes:

Notes S1 to S11 Figs. S1 to S13
