# Supplementary material for: Reconfigurable high-dimensional synthetic photonic lattices
Source: Sci Adv. 2025 Jul 18;11(29):eadw7198. doi: 10.1126/sciadv.adw7198 (PMC13155535; doi:10.1126/sciadv.adw7198)
Supplement: Supplementary file 1 — Notes S1 to S11 Figs. S1 to S13 [file sciadv.adw7198_sm.pdf]

Science Advances
AAAS

Supplementary Materials for

Reconfigurable high-dimensional synthetic photonic lattices

Haiqi Huang *et al.*

Corresponding author: Xiaoyong Hu, xiaoyonghu@pku.edu.cn; Kun Liao, kunliao@pku.edu.cn

Sci. Adv. **11**, eadw7198 (2025)
DOI: 10.1126/sciadv.adw7198

This PDF file includes:

Notes S1 to S11
Figs. S1 to S13

Supplementary Note 1: Derivation of dynamics for temporal synthetic lattice

The complex amplitude of an optical pulse propagating in a fiber can be analogized to the wave function of an electron in a crystal lattice. The pulse propagating in the site ring can be represented as:

$$\psi_0(nT + x\Delta T_x + y\Delta T_y + z\Delta T_z + \cdots + N\Delta T_N) = \psi_0(n, x, y, z, \ldots, N) \tag{S1}$$

Here, N denotes the total number of dimensions. In the experiment, we demonstrated the system in three-dimensional, but this approach can be infinitely extended. Similarly, the pulse on the link ring of the n-th dimension is described by $\psi_m(n, x, y, z, \ldots, N)$, where the superscript m indicates that the pulse is associated with the n-th dimension's link ring. The dynamic process of the first coupling between the site ring and the x-link ring in the n-th period is as follows:

$$\begin{aligned} \psi_0\left(n + \frac{1}{2N}, x, y, z, \ldots, N\right) &= t\psi_0(n, x, y, z, \ldots, N) + \kappa\psi_x(n, x+1, y, z, \ldots, N) \\ \psi_x\left(n + \frac{1}{2N}, x, y, z, \ldots, N\right) &= t\psi_x(n, x, y, z, \ldots, N) + \kappa^*\psi_0(n, x-1, y, z, \ldots, N) \end{aligned} \tag{S2}$$

By extension, the dynamic process of the first coupling between the site ring and the link ring of the m-th dimension is as follows:

$$\begin{aligned} \psi_0\left(n + \frac{m}{2N}, \ldots m, \ldots\right) &= t\psi_0\left(n + \frac{m-1}{2N}, \ldots m, \ldots\right) + \kappa\psi_m\left(n + \frac{m-1}{2N}, \ldots m+1, \ldots\right) \\ \psi_m\left(n + \frac{m}{2N}, \ldots m, \ldots\right) &= t\psi_m\left(n + \frac{m-1}{2N}, \ldots m, \ldots\right) + \kappa^*\psi_0\left(n + \frac{m-1}{2N}, \ldots m-1, \ldots\right) \end{aligned} \tag{S3}$$

The dynamic process of the second coupling between the site ring and the link ring of the m-th dimension is governed by:

$$\begin{aligned} \psi_0\left(n + \frac{m+N}{2N}, \ldots m, \ldots\right) &= t\psi_0\left(n + \frac{m+N-1}{2N}, \ldots m, \ldots\right) + \kappa\psi_m\left(n + \frac{m+N-1}{2N}, \ldots m-1, \ldots\right) \\ \psi_m\left(n + \frac{m+N}{2N}, \ldots m, \ldots\right) &= t\psi_m\left(n + \frac{m+N-1}{2N}, \ldots m, \ldots\right) + \kappa^*\psi_0\left(n + \frac{m+N-1}{2N}, \ldots m+1, \ldots\right) \end{aligned} \tag{S4}$$

Here, it should be noted that the length difference between the site ring and the link ring between the two couplers must be equal, meaning that the lengths of the site ring and all link rings must be consistent. Due to the finite length constraints, the number of lattice points in each dimension of the system cannot be extended infinitely. In the case of the considered evolution step count N_t, the following condition needs to be satisfied:

$$\Delta T_x \ll \Delta T_y \ll \cdots \ll \Delta T_N \ll T \tag{S5}$$

The scaling relationship that needs to be satisfied here is measured by N_t. When these conditions are satisfied, the synthetic lattice experienced by the wave packet within the considered time range becomes indistinguishable from an infinite space. Consequently, band theory can still be applied to describe the wave packet dynamics during this time period.

We have created schematic diagrams illustrating the processes of the one-dimensional and two-dimensional synthetic lattices, respectively, to facilitate a better understanding of the configurations we have employed.

Fig. S1A is a schematic diagram of the optical fiber system, where the red rings and blue rings represent the site ring and the link rings, respectively. The length of the red ring is evenly divided into $\frac{L}{2}$ by two couplers, while the length on the left side of the blue ring is $\frac{L}{2} + \Delta L_x$, and the length on the right side is $\frac{L}{2} - \Delta L_x$. In the site ring, there is also a coupler to couple the external input light into the system and to couple the light out for detection. Below the schematic of the optical fiber,

there is a diagram of the real-space coupled ring array, with the site ring indicated in red and the link rings in blue. The light propagates counterclockwise in the site ring, while it propagates clockwise in the link rings. Fig. S1B shows the process of the pulse starting to propagate in the ring. Fig. S1C shows the result of coupling after the pulse passes through a coupler. Fig. S1D shows the results before and after the pulse reaches and passes through the second coupler. Since the length of the optical fiber on the left side of the link ring is greater than that of the site ring, the light pulse in the link ring reaches the site ring with a delay of ΔT_x. The inset below each diagram show how the pulse propagates in the real-space coupled ring array. The propagation of the pulse in the optical fiber ring follows the same pattern as the propagation in the real-space coupled ring array. Fig. S1E and F show the propagating process of the pulse in the site ring and the link rings during the second cycle.

Furthermore, we present a comparison diagram of the light pulse evolution in a two-dimensional synthetic lattice formed by optical fiber rings and the pulse evolution in a two-dimensional coupled ring array to provide a more detailed explanation of our experimental setup. Fig. S2A shows the schematic diagram of the experimental setup and the equivalent real-space coupled ring array. The red ring represents the site ring, the blue ring represents the x-link ring, and the green ring represent the y-link ring. The lengths of the x-link ring between the two couplers are $\frac{L}{2} - \Delta L_x$ and $\frac{L}{2} + \Delta L_x$, respectively. The lengths of the y-link ring between the two couplers are $\frac{L}{2} - \Delta L_y$ and $\frac{L}{2} + \Delta L_y$, respectively. Fig. S2B to F illustrate the dynamic process of a pulse propagating in the optical fiber rings. The dynamics of the pulse in the real-space array are shown in the real-space coupled ring array diagram on the right side of the optical fiber schematic.

Supplementary Note 2: Pseudocode for data processing

Here, the pseudocode for data processing is provided, which illustrates the process of handling three-dimensional data.

```
inten=zeros(Nx,Ny,Nz,Nt);% Used to record the intensity at the grid.
for i=1:Nt
  measure1=data(startpoint+1+(i-1)*T:startpoint+(i*T));
  for idx_z=1:Nz
    measure2=measure1(start_z+1+(idx_z-1)*dz:start_z+(idx_z)*dz);
    for idx_y=1:Ny
      measure3=measure2(start_y+1+(idx_y-1)*dy:start_y+(idx_y)*dy);
      for idx_x=1:Nx
        measure4=measure3(start_x+1+(idx_x-1)*dx:start_x+(idx_x)*dx);
        inten(idx_x,idx_y,idx_z,i)=max(measure4);
      end
    end
  end
end
```

The evolution process of 3D nonreciprocal coupling is used as an example to demonstrate the data processing procedure. The entire detection sequence is shown in Fig. S3A. Taking the data from the last cycle as an example, it is extracted as shown in Fig. S3B. These pulses correspond to different values of z. By selecting a series of pulses at the same z value, the resulting data

corresponds to different values of y, as shown in Fig. S1C. This process is repeated to obtain pulses corresponding to different values of x shown in Fig. S1D.

Supplementary Note 3: Generalized Brillouin zone

Consider a Su-Schrieffer-Heeger (SSH) model with non-reciprocal coupling, whose Hamiltonian is given by:

$$H(x) = \sum\left[(t_1 + \Delta)c_{i,A}^{\dagger}c_{i,B} + t_2 c_{i,A}^{\dagger}c_{i-1,B}\right] + \sum\left[(t_1 - \Delta)c_{i,B}^{\dagger}c_{i,A} + t_2 c_{i-1,B}^{\dagger}c_{i,A}\right] \quad \text{(S6)}$$

where t_1 and t_2 represent intracell and intercell coupling strengths, respectively. By transforming the Hamiltonian to momentum space:

$$H(k) = \begin{bmatrix} 0 & (t_1 + \Delta) + t_2 e^{-ik} \\ (t_1 - \Delta) + t_2 e^{ik} & 0 \end{bmatrix} \quad \text{(S7)}$$

The corresponding eigenvalues are given by:

$$E(k)^2 = (t_1^2 - \Delta^2) + t_2^2 + 2t_1 t_2 \cos k + 2i\Delta t_2 \sin k$$

The energy band closes when $k = \pi$ and $t_1 = t_2 \pm \Delta$. However, calculating the energy spectrum in real space yields a different result. By applying a similarity transformation to the Hamiltonian:

$$\widetilde{H}(x) = S^{-1}H(x)S \quad \text{(S8)}$$

where $S = diag\{1, r, r^2, \dots, r^N\}$, $r = \sqrt{\left|\frac{t_1-\Delta}{t_1+\Delta}\right|}$.

$$\widetilde{H}(x) = \sum\left[\sqrt{(t_1^2 - \Delta^2)}c_{i,A}^{\dagger}c_{i,B} + t_2 c_{i,A}^{\dagger}c_{i-1,B}\right] + h.c. \quad \text{(S9)}$$

The condition for the band gap closing is $t_1^2 - \Delta^2 = t_2^2$. The bulk eigenstate of $\widetilde{H}(x)$ corresponds to a Bloch wave of $H(k)$, but the corresponding Bloch wavevector will change to $e^{ik'x} = r^x e^{ikx} = e^{i(k - i\log r)x}$, this is called to be Generalized Brillouin zone (GBZ).

For the one-dimensional Floquet Hamiltonian in our system, the evolution matrix is given by:

$$U_1(k) = \begin{bmatrix} t_1 & \kappa_1 \\ e^{\gamma}\kappa_1 & e^{\gamma}t_1 \end{bmatrix}$$

$$U_2(k) = \begin{bmatrix} t_2 & \kappa_2 e^{ik} \\ e^{-\gamma+ik}\kappa_2 & e^{-\gamma}t_2 \end{bmatrix} \quad \text{(S10)}$$

$$U(k) = U_2(k)U_1(k)$$

where $\kappa_{1,2} = i\sqrt{1 - t_{1,2}^2}$. The GBZ in this case takes the form $e^{ik'x} == e^{i(k+ig)x}$.

Supplementary Note 4: Determination of topological phase diagram

The eigenvalues and energy bands of the system can be solved using the transfer matrix methods. The Dirac points emerge at the quasi-energy of π and $k_x = k_y = 0$ during the topological phase transitions. The transformation matrices are given by:

$$M_x = \frac{e^{\gamma}}{i\sin\beta}\begin{bmatrix} 1 & -\cos\beta \\ \cos\beta & -1 \end{bmatrix}$$

$$M_y = \frac{i}{\cos\beta}\begin{bmatrix} 1 & -\sin\beta\, e^{-\gamma} \\ \sin\beta\, e^{\gamma} & -1 \end{bmatrix} \quad \text{(S11)}$$

The existence condition for quasi-energy solutions requires:

$$\left|M_x M_y - I_2\right| = 0 \quad \text{(S12)}$$

The following algebraic relation can be obtained:

$$\sin(2\beta)\cosh(\gamma) = 1 \tag{S13}$$

This result holds under PBC. However, for OBC, the result is different. The equation for solving quasi-energy band is:

$$e^{ik_x}\begin{bmatrix} b_{i,1} \\ d_{i,1} \\ \vdots \\ b_{i,N} \\ d_{i,N} \end{bmatrix} = \begin{bmatrix} M_x & \cdots & 0 \\ \vdots & \ddots & \vdots \\ 0 & \cdots & M_x \end{bmatrix} \begin{bmatrix} e^{-2i\phi} & & & & 0 \\ & M_y & \cdots & 0 & \\ & \vdots & \ddots & \vdots & \\ & 0 & \cdots & M_y & \\ & & & & e^{2i\phi} \end{bmatrix} \begin{bmatrix} b_{i,1} \\ d_{i,1} \\ \vdots \\ b_{i,N} \\ d_{i,N} \end{bmatrix} \tag{S14}$$

Introducing similarity transformation $S = diag\{1,1,e^{\gamma},e^{\gamma} \dots, e^{N\gamma}, e^{N\gamma}\}$, then $\left(\tilde{b}_{i,y}, \tilde{d}_{i,y}\right) = e^{-y\gamma}\left(b_{i,y}, d_{i,y}\right)$, the condition for the phase transition is:

$$\left|\frac{e^{-\gamma}}{\sin\beta\cos\beta}\begin{bmatrix} 1-\sin\beta\cos\beta & \cos\beta-\sin\beta \\ \cos\beta-\sin\beta & 1-\sin\beta\cos\beta \end{bmatrix} - I\right| = 0 \tag{S15}$$

The solution is:

$$1-\sin(\beta)\cos(\beta)\,(e^{\gamma}+1) = \pm\cos(\beta) \mp \sin(\beta) \tag{S16}$$

Supplementary Note 5: Simulation of lattice Hamiltonian

The proposed method can simulate the Floquet model and lattice Hamiltonian model simultaneously. As an example, consider a one-dimensional system where the upper and lower halves of the link ring have a phase difference with respect to the site ring, denoted as $\frac{\pi}{2}-\phi$ and $\frac{\pi}{2}+\phi$. The total phase difference between the link ring and the site ring needs to be π. The dynamic process of site ring is governed by:

$$\begin{cases} \psi\left(x, T+\frac{1}{2}\right) = t\psi(x,T) + i\kappa\psi_x(x,T)e^{i\left(-\phi+\frac{\pi}{2}\right)} \\ \psi_x\left(x, T+\frac{1}{2}\right) = t\psi_x(x,T)e^{i\left(-\phi+\frac{\pi}{2}\right)} + i\kappa\psi(x,T) \end{cases} \tag{S17}$$

$$\begin{cases} \psi(x,T+1) = t\psi\left(x,T+\frac{1}{2}\right) + i\kappa\psi_x\left(x-1,T+\frac{1}{2}\right)e^{i\left(\phi+\frac{\pi}{2}\right)} \\ \psi_x(x,T+1) = t\psi_x\left(x,T+\frac{1}{2}\right)e^{i\left(\phi+\frac{\pi}{2}\right)} + i\kappa\psi\left(x+1,T+\frac{1}{2}\right) \end{cases} \tag{S18}$$

$$\begin{aligned} \psi(x,T+1) = t&\left[t\psi(x,T) + i\kappa\psi_x(x,T)e^{i\left(-\phi+\frac{\pi}{2}\right)}\right] \\ &+ i\kappa\left[t\psi_x(x-1,T)e^{i\left(-\phi+\frac{\pi}{2}\right)} + i\kappa\psi(x-1,T)\right]e^{i\left(\phi+\frac{\pi}{2}\right)} \end{aligned} \tag{S19}$$

$$\begin{aligned} \psi(x,T+1) = t^2\psi(x,T) &+ i\kappa t\left[t\psi_x\left(x,T-\frac{1}{2}\right)e^{i\left(\phi+\frac{\pi}{2}\right)} + i\kappa\psi\left(x+1,T-\frac{1}{2}\right)\right]e^{i\left(-\phi+\frac{\pi}{2}\right)} \\ -i\kappa^2\psi(x-1,T)e^{-i\phi} &- \kappa t\left[t\psi_x\left(x-1,T-\frac{1}{2}\right)e^{i\left(\phi+\frac{\pi}{2}\right)} + i\kappa\psi\left(x,T-\frac{1}{2}\right)\right] \end{aligned} \tag{S20}$$

Due to $|\psi_x| \ll |\psi|$, $t\psi\left(x,T-\frac{1}{2}\right) \approx \psi(x,T)$ and $\kappa = \sqrt{1-t^2}$ and $t \to 1$.

$$\psi(x,T+1) - \psi(x,T) \approx -i\kappa^2\left[e^{-i\phi}\psi(x-1,T) + e^{i\phi}\psi(x+1,T)\right] \tag{S21}$$

The effective Hamiltonian is:

$$H_{eff} = \kappa^2\sum_x |x\rangle\langle x-1|e^{-i\phi} + h.c. \tag{S22}$$

For different values of x, ϕ can take different values, which can be controlled through a phase modulator. The derivation process for higher-dimensional models is similar.

The Hamiltonian of Hatano-Nelson model can also be simulated using this approach. Assume that the light propagating in the positive direction through the link ring gains amplification. The evolution is governed by:

$$\psi(x,T+1) = t^2\psi(x,T) - i\kappa^2 t e^{\gamma}\psi(x-1,T) - i\kappa^2 t e^{-\gamma}\psi(x+1,T) \qquad (S23)$$

Here t is the coupling coefficient of fiber coupler and $\kappa = \sqrt{1-t^2}$ in the ideal case. By setting $t \to 1$, the equation is approximately equal to:

$$\psi(x,T+1) - \psi(x,T) \approx -i\kappa^2[e^{\gamma}\psi(x-1,T) + e^{-\gamma}\psi(x+1,T)] \qquad (S24)$$

The corresponding effective Hamiltonian is:

$$H_{eff} = \kappa^2 \sum_x |x\rangle\langle x-1|e^{-\gamma} + |x\rangle\langle x+1|e^{\gamma} \qquad (S25)$$

For comparison, the Hatano-Nelson Hamiltonian is given by:

$$H_{HN} = \sum_x J_R|x\rangle\langle x-1| + J_L|x\rangle\langle x+1| \qquad (S26)$$

By introducing $\kappa^2 = \sqrt{J_L J_R}$ and $e^{\gamma} = \sqrt{J_L/J_R}$, the effective Hamiltonian becomes equivalent to the Hatano-Nelson Hamiltonian.

Next, we consider introducing a polarization controller in the link ring to realize spin-orbit coupling. Considering using two different linear polarization directions in the optical fiber as pseudospins. In a single-mode fiber, these directions correspond to the fast axis and the slow axis. We assume that the refractive index difference is small enough that it does not lead to the walk-off effect. The transformer matrix of polarization controller is:

$$M(\theta,\alpha,\beta) = \begin{bmatrix} e^{i\alpha} & 0 \\ 0 & 1 \end{bmatrix} \begin{bmatrix} \cos\theta & -sin\theta \\ sin\theta & \cos\theta \end{bmatrix} \begin{bmatrix} 1 & 0 \\ 0 & e^{i\beta} \end{bmatrix} \qquad (S27)$$

$$\psi(x,T+1) - \psi(x,T) \approx -i\kappa\left[e^{-i\phi}M(\theta,\alpha,\beta)\psi(x-1,T) + e^{i\phi}M(\theta,-\alpha,-\beta)\psi(x+1,T)\right] \qquad (S28)$$

Here $\psi = \left[\psi_{slow}, \psi_{fast}\right]^T$. For example, $\theta = \frac{\pi}{2}, \alpha+\beta = \pi$, the Hamiltonian is:

$$H_{eff} = \kappa^2 \sum_x |x\rangle\langle x-1|e^{-i\phi} \otimes \sigma_x + h.c. \qquad (S29)$$

Take $\theta = \frac{\pi}{2}, \alpha+\beta = 0, \phi = \frac{\pi}{2} + \phi'$, the Hamiltonian is:

$$H_{eff} = \kappa^2 \sum_x |x\rangle\langle x-1|e^{-i\phi'} \otimes \sigma_y + h.c. \qquad (S30)$$

Take $\theta = 0, \alpha = 0, \beta = \pi$, the Hamiltonian is:

$$H_{eff} = \kappa^2 \sum_x |x\rangle\langle x-1|e^{-i\phi'} \otimes \sigma_z + h.c. \qquad (S31)$$

Another approach is to use circular polarization (spin) of light as a pseudospin. In this scheme, a polarizing beam splitter resolves the input circularly polarized light into orthogonal linear components, which are then independently phase-modulated. The transformation process is governed by the following transfer matrix:

$$M(\theta,\alpha,\beta) = \frac{1}{2}\begin{bmatrix} 1 & i \\ i & 1 \end{bmatrix} \begin{bmatrix} e^{i\theta} & 0 \\ 0 & e^{-i\theta} \end{bmatrix} \begin{bmatrix} 1 & i \\ i & 1 \end{bmatrix} \begin{bmatrix} e^{i\alpha} & 0 \\ 0 & e^{-i\beta} \end{bmatrix} \qquad (S32)$$

One can find $M\left(0,-\frac{\pi}{2},\frac{\pi}{2}\right) = \sigma_x$, $M(0,0,\pi) = \sigma_y$ and $M\left(\frac{\pi}{2},-\frac{\pi}{2},\frac{\pi}{2}\right) = \sigma_z$

Multi-mode optical fibers carrying Orbital Angular Momentum (OAM) can be used to implement a four-state system. The modes are denoted as $|l, p\rangle$, where l represents the topological charge and p denotes the polarization direction. The four modes are selected as: $|1, x\rangle, |-1, x\rangle, |1, y\rangle$ and $|-1, y\rangle$. A mode splitter is used to separate different OAM modes from the multi-mode optical fiber into four single-mode fibers. Subsequently, a 4th-order Mach-Zehnder Interferometer (MZI) is employed to construct the $U(4)$ matrix, realizing the required coupling. In addition to OAM, other orthogonal bases can also be used, with the principle remaining the same.

Using the method above, a bulk Hamiltonian can be expressed as:

$$
\begin{aligned}
H = {} & t_1\left(\sum_{x,y,z} |x+1, y, z\rangle\langle x, y, z|\, \sigma_x + h.c.\right) \\
& + t_1\left(\sum_{x,y,z} |x, y+1, z\rangle\langle x, y, z|\, \sigma_y + h.c.\right) \\
& + \left(\sum_{x,y,z} (t_3|x, y, z+1\rangle\langle x, y, z| + m)\sigma_z + h.c.\right)
\end{aligned} \quad (S33)
$$

The momentum space form of this Hamiltonian is given by Equation (5) in the manuscript. Using OAM as basis, the Hamiltonian of a four-band system is given by:

$$
\begin{aligned}
H = {} & t_1\left(\sum_{x,y,z,r_4,r_5} |x+1, y, z, r_4, r_5\rangle\langle x, y, z, r_4, r_5|\, \sigma_x \otimes \sigma_0 + h.c.\right) \\
& + t_1\left(\sum_{x,y,z,r_4,r_5} |x, y, z, r_4, r_5\rangle\langle x, y+1, z, r_4, r_5|\, \sigma_y \otimes \sigma_0 + h.c.\right) \\
& + t_1\left(\sum_{x,y,z,r_4,r_5} |x, y, z, r_4, r_5\rangle\langle x, y, z+1, r_4, r_5|\, \sigma_z \otimes \sigma_x + h.c.\right) \\
& + t_1\left(\sum_{x,y,z,r_4,r_5} |x, y, z, r_4, r_5\rangle\langle x, y, z, r_4+1, r_5|\, \sigma_z \otimes \sigma_y + h.c.\right) \\
& + \left(\sum_{x,y,z,r_4,r_5} (t_5|x, y, z, r_4, r_5\rangle\langle x, y, z, r_4, r_5+1| + m)\sigma_z \otimes \sigma_0 + h.c.\right) \\
& + \left(\sum_{x,y,z,r_4,r_5} \left(\frac{1}{2} i\gamma |x, y, z, r_4, r_5\rangle\langle x, y, z, r_4, r_5|\right)[\Gamma_{31}, \Gamma_{32}] + h.c.\right)
\end{aligned} \quad (S34)
$$

By performing a Fourier transform in space, we can obtain Equation (7) in the manuscript, thereby realizing the Weyl surfaces.

Supplementary Note 6: Theoretical calculation results

We presented the theoretically computed evolutionary results for comparison with the experimental measurements, as shown in Fig. S4-S6. While the experimental results align with the theoretical trends, discrepancies remain. These deviations may originate from multiple factors: (1) temperature drift affecting the EDFA gain coefficient, and (2) noise impacting the signal-to-noise ratio. When the system operates in the PT-symmetry breaking phase, both the signal and noise grow exponentially, leading to a significantly reduced signal-to-noise ratio. To mitigate this issue, we applied an overall shift towards loss in the system, thereby preventing excessive amplification of noise.

We computed the mode distribution of the eigenstate corresponding to the maximum imaginary part in a 9×9 array in the experiment of periodic temporal modulation, as shown in Fig. S7. Fig. S7A displays the intensity distribution of the eigenstate with the maximum imaginary part on the lattice ring, while Fig. S7B shows the result after the state has evolved for half a period. It can be observed that the intensity on the lattice ring significantly weakens, which is consistent with our theoretical explanation.

Supplementary Note 7: Evolution-based analysis of band structure

The band structure can be analyed through the spectrum obtained by applying two-dimensional Fourier transform to the wave function. Since the measured result here provide the intensity information of the evolutionary process, phase information can only be obtained by referencing the interference between the light and the signal light. However, we can leverage prior knowledge of the phase. From the evolution matrix, we know that the wave function within the site rings remains real throughout the evolution. Therefore, we can directly obtain the wave function of the site ring by taking the square root. Performing discrete Fourier transform on $\psi(r,t)$:

$$\tilde{\psi}(E,k) = F \circ \psi(r,t) = \frac{1}{\sqrt{N_t N_r}} \sum_{r,t} \psi(r,t) \exp(-ikr + iEt) \qquad (S35)$$

We have:

$$\psi(r,t) = \frac{1}{\sqrt{N_k}} \sum_{k} \psi(k,t) \exp(ikr) = \frac{1}{\sqrt{N_k}} \sum_{k} \psi(k,0) \exp(ikr - iE_k t) \qquad (S36)$$

When $E = real(E_k)$, $\left|\tilde{\psi}(E,k)\right|^2$ reaches maximum value. Thus, by examining the distribution of $\left|\tilde{\psi}(E,k)\right|^2$, we can determine the band structure. We verify this in 1D nonreciprocal coupling system, and the results are shown in Fig. S8. The experimental measurements are in excellent agreement with the theoretical predictions. Due to the influence of the imaginary part of the energy bands, the gain component becomes apparent in this context. The real part of the theoretical energy band is shown, with the color representing the imaginary part of the energy band. Fig. S8 illustrates non-reciprocal coupling in the negative x-direction and positive x-direction, respectively.

Supplementary Note 8: Experimental Details

In the experiment, it is necessary to ensure that the total lengths of the site ring and the link ring are identical. When calculating the lengths, the phase modulators, amplitude modulators, EDFAs, and filters on both the site and link rings are considered to have the same length, and therefore, are not included in the total length calculation. The coupling device on the site ring, which is externally coupled, has a length of 3 meters. Its role is to inject the incident pulse into the site ring and couple

part of the pulse to the photodetector. A similar coupling device is introduced on the link ring, not only to balance the total length but also to measure the pulse within the link ring.

For the 1D case, the length difference is set to 25 meters. In the 2D case, it is important to note that the site ring undergoes four couplings during the coupling process, while each link ring only experiences two. Therefore, an additional 6 meters must be added to the link ring to account for this difference. The length differences of the two link rings are 3 meters and 38 meters, respectively. For the 3D case, the link rings need to be adjusted by an additional 12 meters. The length differences for the three link rings are 7 meters, 82 meters, and 1,000 meters, respectively. Fig. 1B shows the experiment schematic for 2D case. A laser with a wavelength of 1550 nm emits continuous wave. The system employs a 1550 nm continuous-wave laser source whose output is modulated into pulsed signals using an intensity modulator driven by an arbitrary waveform generator. The site ring and the link ring are interconnected via 50/50 couplers. The output signal detected by a photodetector (PD), which converts the electrical signal to the oscilloscope for display.

To aid in a better understanding, we present the mathematical form of the signal generated by the signal generator required to implement the 2D funnel state. The transmittance of the modulator can be written as: $T = T_0 + T_m \cos\left(\pi \frac{V - V_0}{V_\pi}\right)$, where V_h denotes the voltage at high transmittance ($T = 1$), which corresponds to the gain, and V_l represents the voltage at low transmittance, which corresponds to the loss. The signal expressions for the cases of one-dimensional and two-dimensional nonreciprocal coupling are presented first. Since at this case, the conditions of all the x-link rings are identical, and the conditions of y-link rings are also identical.

$$V_{x(y)} = \begin{cases} V_h & \text{gain} \\ V_l & \text{loss} \end{cases} \tag{S37}$$

It is important to note that for different modulators, the value of V required to achieve the same transmittance may vary.

Then, we take the case of realizing two-dimensional funnel state as another example to show reconfigurability of the scheme. The expression for the signal of the intensity modulator on the y-link ring is given by:

$$V_y(t) = \begin{cases} V_l & nT < t < nT + y_f \Delta T_y \\ V_h & nT + y_f \Delta T_y < t < (n+1)T \end{cases} \tag{S38}$$

where y_f represents the temporal position of the funnel state on the y-axis.

The expression for the signal of the intensity modulator on the x-link ring is given by:

$$V_x(t) = \begin{cases} V_l & nT + m\Delta T_y < t < nT + m\Delta T_y + x_f \Delta T_x \\ V_h & nT + m\Delta T_y + x_f \Delta T_x < t < nT + (m+1)\Delta T_y \end{cases} \tag{S39}$$

where x_f represents the temporal position of the funnel state on the x-axis.

Supplementary Note 9: Extended experimental results

Due to the limitations of image size for the manuscript, the 3D experimental results are only presented at $T = 1, 3, 5,$ and 7. Here, we provide the corresponding experimental results for $T = 2, 4,$ and 6, as shown in Fig. S9.

The extension of physical systems into higher dimensions enables the manifestation of unique phenomena exclusive to high-dimensional spaces. We focus on reconfigurable non-Hermitian routing channels as a representative case. In 1D systems, information transmission is constrained to a singular fixed pathway, whereas higher-dimensional architectures exhibit abundant pathway

selection characteristics. Through implementation of gain-loss modulation strategies, we successfully constructed non-Hermitian transmission channels with path reconfigurability, achieving directional information transfer along distinct geometric pathways.

Fig. S8 shows the schematic diagram and results of non-Hermitian router. Fig. S10A illustrates the engineering principle of directional non-Hermitian router, establishing a controllable photon transport channel between start and end sites. Fig. S10B shows the experimental observations of state evolution along this designated pathway. For comparative analysis, Fig. S10C presents an alternative transmission path configuration with distinct features, whose corresponding dynamical evolution process is depicted in Fig. S10D.

Supplementary Note 10: Geometry-dependent skin effect

When the dimensionality of synthetic lattices is increased to higher dimensions, phenomena that are not present in lower-dimensional systems may emerge. High-dimensional systems exhibit more intricate boundary properties, with one of the most notable examples being the geometry-dependent skin effect. This effect refers to the phenomenon in non-Hermitian systems, where the manifestation of skin effects is intricately linked to the geometry of the system's boundary. We propose a specific scheme to realize the geometry-dependent skin effect within our system and validate it through numerical simulations.

To begin, we construct a two-dimensional lattice that demonstrates the geometry-dependent skin effect. The Hamiltonian of the system is:

$$H(t) = \sum_{i,j} \begin{cases} \kappa_{x1} c_{i,j}^{\dagger} a_{i,j} & nT < t < \left(n + \frac{1}{5}\right)T \\ \kappa_{y1} c_{i,j}^{\dagger} b_{i,j} & \left(n + \frac{1}{5}\right)T < t < \left(n + \frac{2}{5}\right)T \\ \kappa_{x2} c_{i,j}^{\dagger} a_{i-1,j} & \left(n + \frac{2}{5}\right)T < t < \left(n + \frac{3}{5}\right)T + h.c \\ \kappa_{y2} c_{i,j}^{\dagger} b_{i,j-1} & \left(n + \frac{3}{5}\right)T < t < \left(n + \frac{4}{5}\right)T \\ (-1)^{i+j} \gamma c_{i,j}^{\dagger} c_{i,j} & \left(n + \frac{4}{5}\right)T < t < (n+1)T \end{cases} \qquad (S40)$$

A schematic diagram of this process is shown in Fig. S11A. We set $\exp\left(\frac{1}{5}\gamma T\right) = \frac{3}{2}$ and calculated the eigenstates within a square region, summing their squared norms to obtain the eigenstate distribution, as shown in Fig. S11B. It can be observed that the eigenstate distribution does not exhibit the skin effect. Next, we divided the square into two right triangular regions along the diagonal, and as depicted in Fig. S11C, the skin effect appears distinctly in both regions. We then computed the eigenstate distributions for a triangular region and a concave pentagonal region, as shown in Fig. S11D. The results indicate that the skin effect also manifests separately in each of these two regions.

Supplementary Note 11: Dynamical Evolution in High-Dimensional Systems

Temporal synthetic lattice systems offer a versatile platform for exploring dynamical phenomena in high-dimensional systems. In this work, we demonstrate this approach through the example of the four-dimensional quantum Hall effect.

We consider a four-dimensional synthetic lattice system comprising a site ring and four link rings. The dynamics are governed by:

$$\begin{cases} \psi\left(x,y,z,w;T+\frac{1}{8}\right) = t\psi(x,y,z,w;T) + i\kappa\psi_x\left(x-\frac{1}{2},y,z,w;T\right) \\ \psi_x\left(x-\frac{1}{2},y,z,w;T+\frac{1}{8}\right) = t\psi_x\left(x-\frac{1}{2},y,z,w;T\right) + i\kappa\psi(x,y,z,w;T) \end{cases} \tag{S41}$$

$$\begin{cases} \psi\left(x,y,z,w;T+\frac{2}{8}\right) = t\psi\left(x,y,z,w;T+\frac{1}{8}\right) + i\kappa\psi_y\left(x,y-\frac{1}{2},z,w;T+\frac{1}{8}\right) \\ \psi_y\left(x,y-\frac{1}{2},z,w;T+\frac{2}{8}\right) = t\psi_y\left(x,y-\frac{1}{2},z,w;T+\frac{1}{8}\right) + i\kappa\psi\left(x,y,z,w;T+\frac{1}{8}\right) \end{cases} \tag{S42}$$

The dynamics in the z and w directions are similar to x and y. For the second round of coupling, the site ring will couple once with the link ring in the positive direction for each dimension.

$$\begin{cases} \psi\left(x,y,z,w;T+\frac{5}{8}\right) = t\psi\left(x,y,z,w;T+\frac{4}{8}\right) + i\kappa\psi_x\left(x+\frac{1}{2},y,z,w;T+\frac{4}{8}\right) \\ \psi_x\left(x+\frac{1}{2},y,z,w;T+\frac{5}{8}\right) = t\psi_x\left(x+\frac{1}{2},y,z,w;T+\frac{4}{8}\right) + i\kappa\psi\left(x,y,z,w;T+\frac{4}{8}\right) \end{cases} \tag{S43}$$

$$\begin{cases} \psi\left(x,y,z,w;T+\frac{6}{8}\right) = t\psi\left(x,y,z,w;T+\frac{5}{8}\right) + i\kappa\psi_y\left(x,y+\frac{1}{2},z,w;T+\frac{5}{8}\right) \\ \psi_y\left(x,y+\frac{1}{2},z,w;T+\frac{6}{8}\right) = t\psi_y\left(x,y+\frac{1}{2},z,w;T+\frac{5}{8}\right) + i\kappa\psi\left(x,y,z,w;T+\frac{5}{8}\right) \end{cases} \tag{S44}$$

Here $\psi_x\left(x+\frac{1}{2},y,z,w;T+\frac{4}{8}\right) = i\psi_x\left(x+\frac{1}{2},y,z,w;T+\frac{1}{8}\right) * \exp(i\phi_x)$ and $\psi_y\left(x,y+\frac{1}{2},z,w;T+\frac{5}{8}\right) = i\psi_y\left(x,y+\frac{1}{2},z,w;T+\frac{2}{8}\right) * \exp(i\phi_y)$. $\phi_x\phi_\mathrm{x}$ and ϕ_y are the gauge-potential. On the other hand, we have $\psi_x\left(x+\frac{1}{2},y,z,w;T\right) = i\psi_x\left(x+\frac{1}{2},y,z,w;T-\frac{3}{8}\right) * \exp(-i\phi_x)$ and $\psi_y\left(x,y+\frac{1}{2},z,w;T+\frac{1}{8}\right) = i\psi_y\left(x,y+\frac{1}{2},z,w;T-\frac{2}{8}\right) * \exp(i\phi_y)$. These are the same for z and w directions.

Our goal is to derive a dynamical equation that contains only the wave function of the site ring, simplifying the system and obtaining its effective Hamiltonian. As an example, we consider the dynamical process of the site ring and link ring in the x-direction. The same principle can be applied to the other directions through linear superposition.

$$\psi(x,y,z,w;T+1) = t^4\psi\left(x,y,z,w;T+\frac{4}{8}\right) + i\kappa t^3\psi_x\left(x+\frac{1}{2},y,z,w;T+\frac{4}{8}\right) \tag{S45}$$

$$\psi\left(x,y,z,w;T+\frac{4}{8}\right) = t^4\psi(x,y,z,w;T) + i\kappa t^3\psi_x\left(x-\frac{1}{2},y,z,w;T\right) \tag{S46}$$

$$\begin{aligned} \psi(x,y,z,w;T+1) = t^8\psi(x,y,z,w;T) + i\kappa t^7\psi_x\left(x-\frac{1}{2},y,z,w;T\right) \\ +i\kappa t^3\psi_x\left(x+\frac{1}{2},y,z,w;T+\frac{4}{8}\right) \end{aligned} \tag{S47}$$

$$\begin{aligned} \psi_x\left(x+\frac{1}{2},y,z,w;T+\frac{4}{8}\right) &= i\psi_x\left(x+\frac{1}{2},y,z,w;T+\frac{1}{8}\right) * \exp(i\phi_x) \\ &= i\exp(i\phi_x)\left[t\psi_x\left(x+\frac{1}{2},y,z,w;T\right) + i\kappa\psi(x+1\,,y,z,w;T)\right] \end{aligned} \tag{S48}$$

$$\psi(x,y,z,w;T+1) = t^8\psi(x,y,z,w;T) + i\kappa t^7\psi_x\left(x-\frac{1}{2},y,z,w;T\right)$$
$$-\kappa t^3\exp(i\phi_x)\left[t\psi_x\left(x+\frac{1}{2},y,z,w;T\right) + i\kappa\psi(x+1\,,y,z,w;T)\right]$$
$$= t^8\psi(x,y,z,w;T) \qquad (S49)$$
$$-\kappa t^7\exp(-i\phi_x)\left[t\psi_x\left(x-\frac{1}{2},y,z,w;T-\frac{1}{2}\right) + i\kappa\psi\left(x-1,y,z,w;T-\frac{1}{2}\right)\right]$$
$$-\kappa t^3\, exp(i\phi_x)\left[t\psi_x\left(x+\frac{1}{2},y,z,w;T\right) + i\kappa\psi(x+1\,,y,z,w;T)\right]$$

Since $t\to 1$, we have the approximation: $t^4\psi\left(x-1,y,z,w;T-\frac{1}{2}\right)\approx\psi(x-1,y,z,w;T)$, $|\psi_x|\ll|\psi|$.

$$\psi(x,y,z,w;T+1)\approx t^8\psi(x,y,z,w;T) - i\kappa^2t^3\exp(-i\phi_x)\,\psi(x-1,y,z,w;T)$$
$$-i\kappa^2t^3\exp(i\phi_x)\,\psi(x+1,y,z,w;T) \qquad (S50)$$

Through the same derivation process, considering the contribution of the y,z,w link ring, the complete dynamic evolution process is:

$$\psi(x,y,z,w;T+1)\approx t^8\psi(x,y,z,w;T) - i\kappa^2t^3\exp(-i\phi_x)\,\psi(x-1,y,z,w;T)$$
$$-i\kappa^2t^3\exp(i\phi_x)\,\psi(x+1,y,z,w;T)$$
$$-i\kappa^2t^3\exp\left(-i\phi_y\right)\psi(x,y-1,z,w;T)$$
$$-i\kappa^2t^3\exp\left(i\phi_y\right)\psi(x,y+1,z,w;T)$$
$$-i\kappa^2t^3\exp(-i\phi_z)\,\psi(x,y,z-1,w;T) \qquad (S51)$$
$$-i\kappa^2t^3\exp(i\phi_z)\,\psi(x,y,z+1,w;T)$$
$$-i\kappa^2t^3\exp(-i\phi_w)\,\psi(x,y,z,w-1;T)$$
$$-i\kappa^2t^3\exp(i\phi_w)\,\psi(x,y,z,w+1;T)$$

By further approximation, the following expression can be obtained:

$$\psi(x,y,z,w;T+1)-\psi(x,y,z,w;T)\approx -i\kappa^2\exp(-i\phi_x)\,\psi(x-1,y,z,w;T)$$
$$-i\kappa^2\exp(i\phi_x)\,\psi(x+1,y,z,w;T)$$
$$-i\kappa^2\exp\left(-i\phi_y\right)\psi(x,y-1,z,w;T)$$
$$-i\kappa^2\exp\left(i\phi_y\right)\psi(x,y+1,z,w;T)$$
$$-i\kappa^2\exp(-i\phi_z)\,\psi(x,y,z-1,w;T) \qquad (S52)$$
$$-i\kappa^2\exp(i\phi_z)\,\psi(x,y,z+1,w;T)$$
$$-i\kappa^2\exp(-i\phi_w)\,\psi(x,y,z,w-1;T)$$
$$-i\kappa^2\exp(i\phi_w)\,\psi(x,y,z,w+1;T)$$

The effective Hamiltonian is given by:

$$H = \kappa^2\textstyle\sum_{i=x,y,z,w}\exp(-i\phi_r)\,|r\rangle\langle r-e_i| + h.c. \qquad (S53)$$

To realize the four-dimensional quantum Hall effect, we adopt the following Hamiltonian:

$$H = H_{xz} + H_{yw}$$
$$= \kappa^2[e^{-ib_{xz}z}|r\rangle\langle r-e_x| + e^{-ib_{yw}w}|r\rangle\langle r-e_y| \qquad (S54)$$
$$+|r\rangle\langle r-e_z| + |r\rangle\langle r-e_w|] + h.c.$$

where $b_{xz} = 2\pi p_{xz}/q_{xz}$, $b_{yw} = 2\pi p_{yw}/q_{yw}$, p,q are coprime positive integers. To investigate the boundary effects of such a system, we construct a supercell. This supercell is periodic in the x, y, and w directions, and has a finite number of lattice points in the z-direction. Note that the

projection of the superlattice in the w-direction contains q_{yw} lattice points. The Hamiltonian's size is $\left(N_z * q_{yw}\right) * \left(N_z * q_{yw}\right)$.

By choosing k_x as the independent variable representing the energy band, and scanning k_y and k_w, we can plot the energy bands. This allows us to obtain information about the bulk states and boundary states in the z-direction. Fig. S12 shows the band structure with k_x as the independent variable, while k_y and k_z are traversed. We choose $N_z = 3$, $p_{xz} = p_{yw} = 1$ and $q_{xz} = q_{yw} = 3$ to calculate the energy bands. The gray points represent eigenstates distributed within the bulk, the red points indicate states located at the lower boundary of z-direction, and the blue points correspond to states at the upper boundary of z-direction.

The regions marked in red and blue represent the states of super-boundary transmission in the z-direction. The size of the theoretical simulation region is $N_x = 20$, $N_y = 10$, $N_z = 12$, $N_w = 10$. The initial state consists of two Gaussian wave packets. In the x-direction, they are localized around $x = 20$, with a momentum of $k_x = 2$ and the wave packet's width is 2. In the y and w directions, the initial values are $y = w = 5$. These two wave packets are located at $z = 1$ and $z = 12$, respectively, in the z-direction.

From the energy band diagram, it can be observed that the edge states at the top have a negative group velocity, while those at the bottom exhibit a positive group velocity. This observation is further confirmed by the wave packet evolution diagrams. The intensity distributions of the wave packet are shown for the evolution at 1 step, 21 steps, and 41 steps, as illustrated in Fig. S13.

Furthermore, if a fixed beam splitter is used, continuous coupling between the site ring and the link ring occurs, which hinders the formation of edge states. Two methods can address this issue. The first is to use a variable beam splitter, allowing dynamic control of the coupling coefficient and thereby interrupting the coupling at the edge of the synthetic lattice. The second method involves constructing a domain wall within the bulk, where a phase difference in the coupling exists on either side of the compensation wall. For example, the phase of the coupling coefficient in the x-direction can be expressed as:

$$\phi_x = \begin{cases} b_{xz}z & z \leq z_0 \\ b_{xz}(z+1) & z > z_0 \end{cases} \qquad (S55)$$

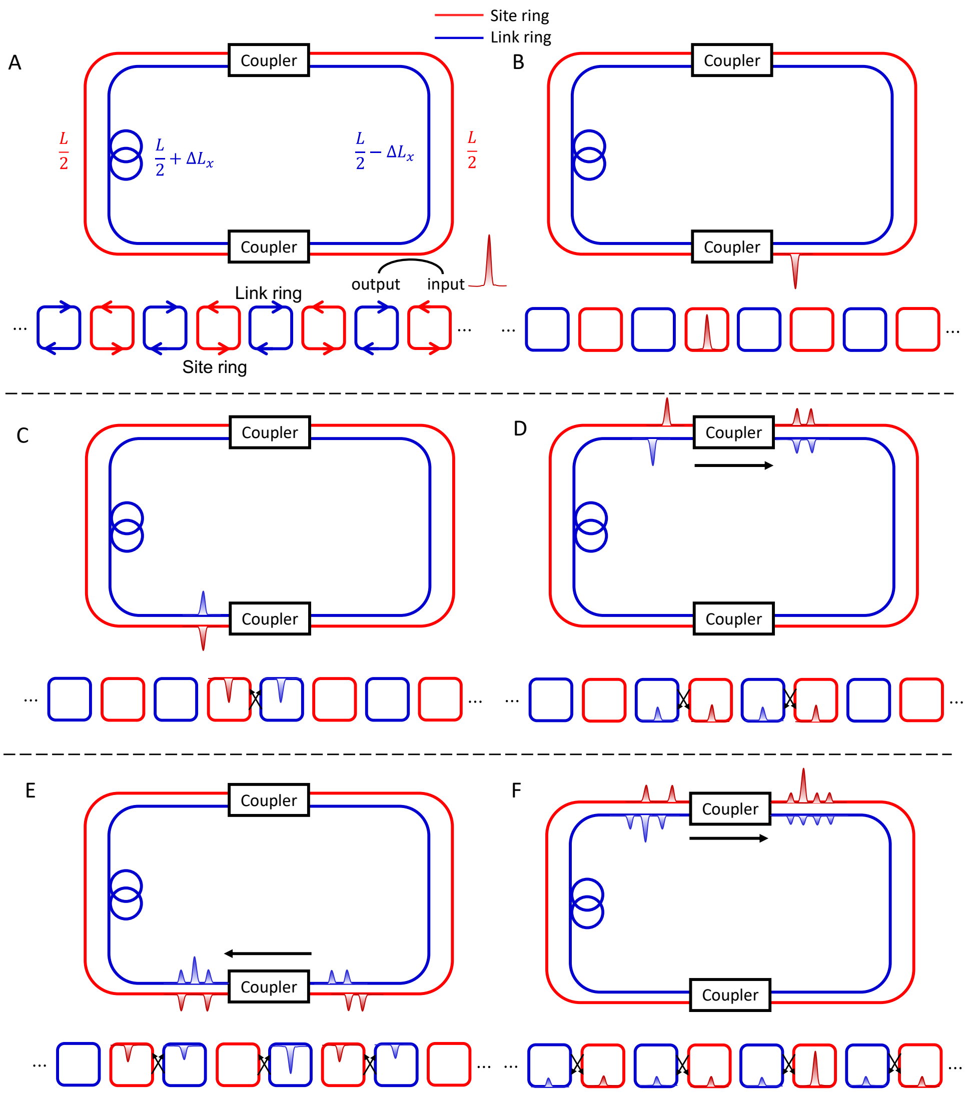

Fig. S1. Comparison of the pulse transmission process in the optical fiber ring and the real-space coupled ring array.

(A) Schematic diagram of the optical fiber ring and the real-space coupled ring array. (B)–(F) Schematic comparison of the pulse evolution process in the optical fiber ring and the coupled ring array.

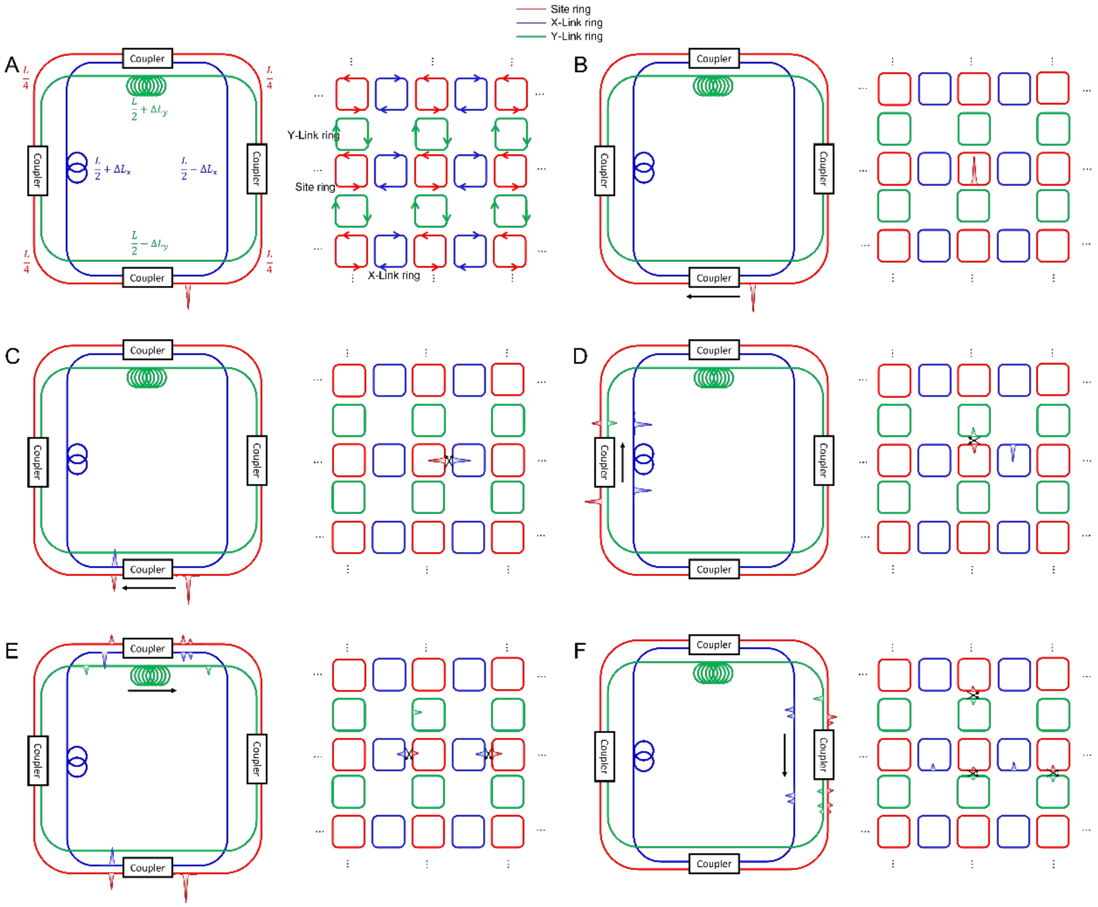

Fig. S2. Comparison between the schematic of the optical fiber ring constructing a two-dimensional synthetic lattice and the real-space coupled ring array.

(A) Schematic diagram of the optical fiber ring constructing a two-dimensional synthetic lattice and the real-space coupled ring array. (B)–(F) Schematic comparison of the pulse evolution process in the optical fiber ring and the coupled ring array.

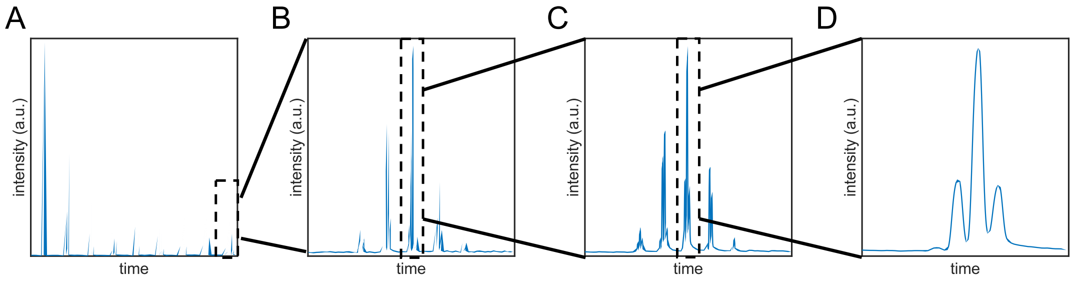

Fig. S3 Schematic of data processing.

(A) The overall pulse sequence. (B) The pulse sequences at different values of z within the same cycle. (C) The pulse sequences at different values of y within the same z. (D) The pulse sequences at different values of x within the same y.

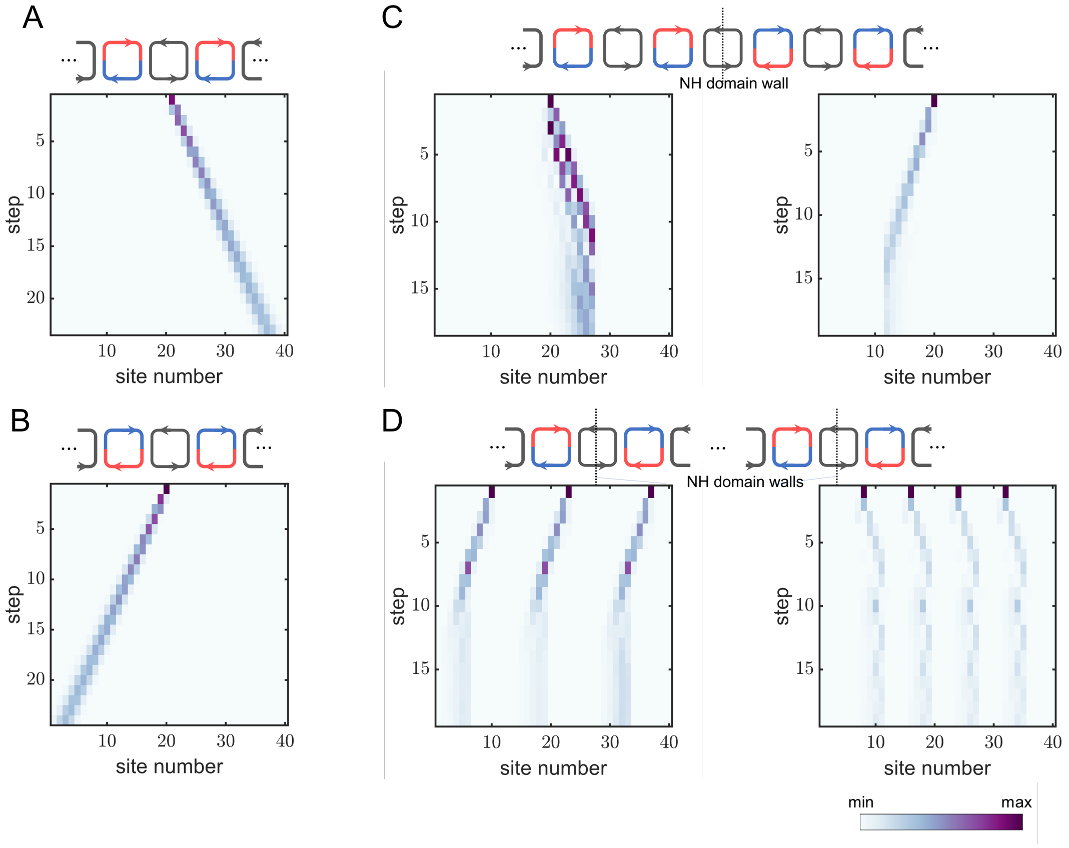

Fig. S4 Theoretical calculation results in 1D space.

(A to B) 1D nonreciprocal coupling evolution results. (C) 1D topological funnel state evolution results. (D) 1D multiple funnel states evolution results.

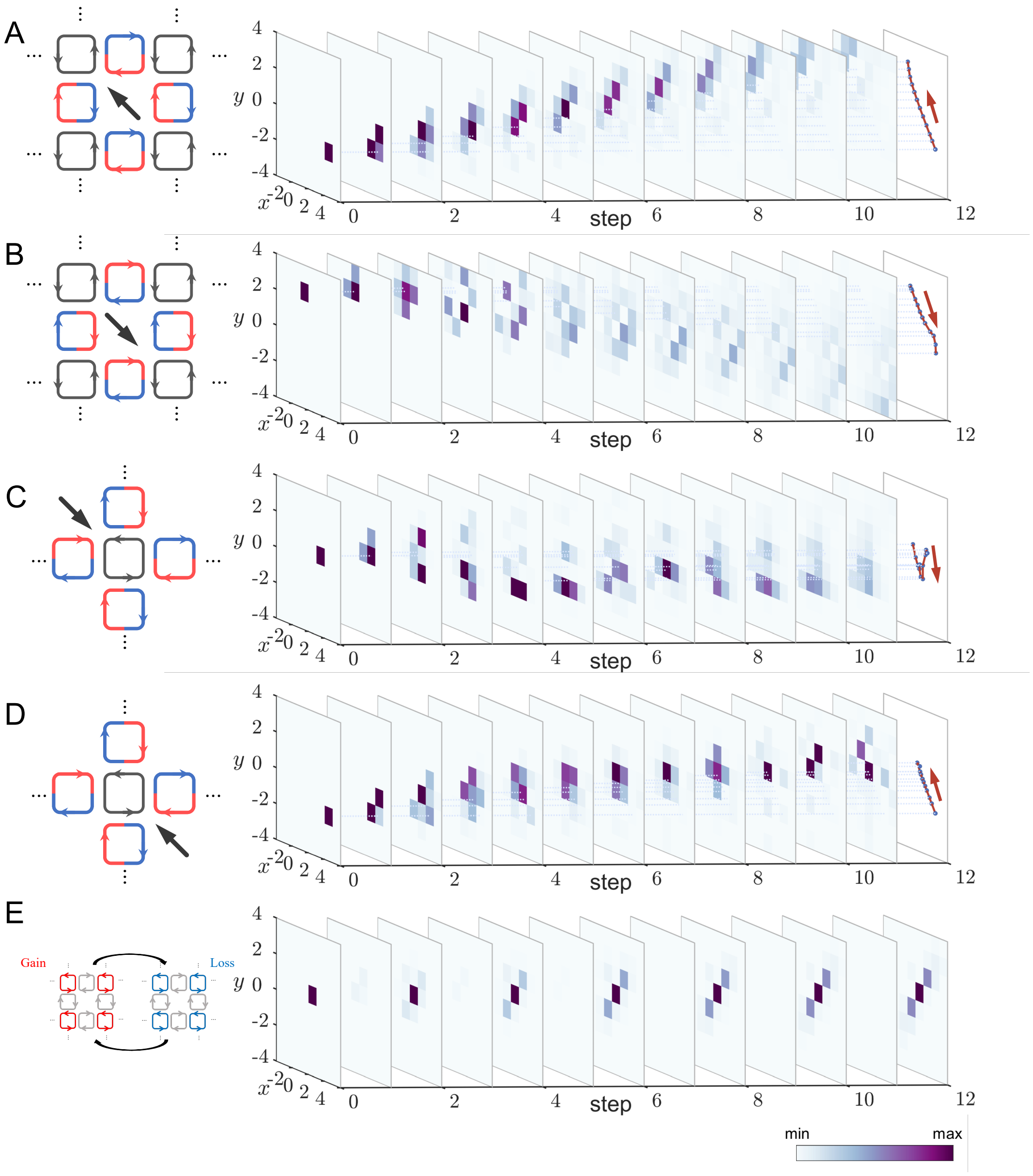

Fig. S5 Theoretical calculation results in 2D space.

(A to B) 2D nonreciprocal coupling evolution results. (C to D) 2D topological funnel state evolution results. (E) Non-Hermitian photonic time crystals evolution results.

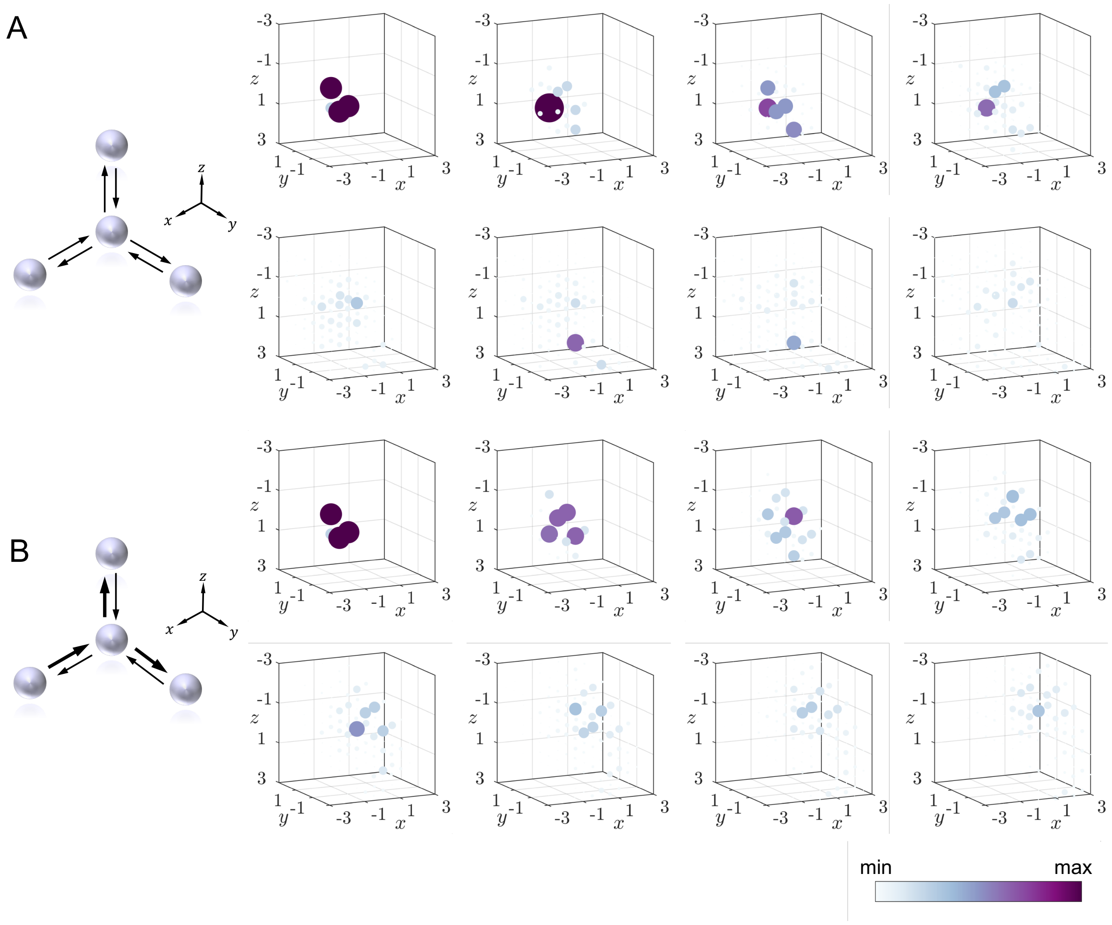

Fig. S6 Theoretical calculation results in 3D space.

(A) 3D reciprocal coupling evolution results for $T = 1, 2 \dots 8$. (B) 3D nonreciprocal coupling evolution results for $T = 1, 2 \dots 8$.

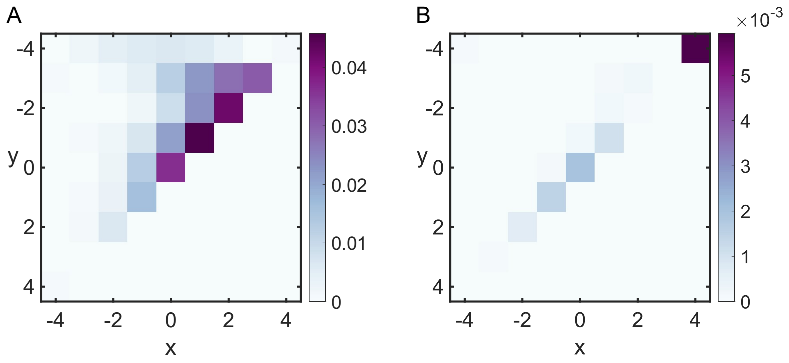

Fig. S7. The profile of the eigenstate with the largest imaginary component.

(A) The profile of eigenstate. The energy is primarily distributed in the site rings. (B) The result after the site ring undergoes gain for half a period. The energy is primarily distributed in the link rings, such that the state loses very little energy during the loss in the site ring.

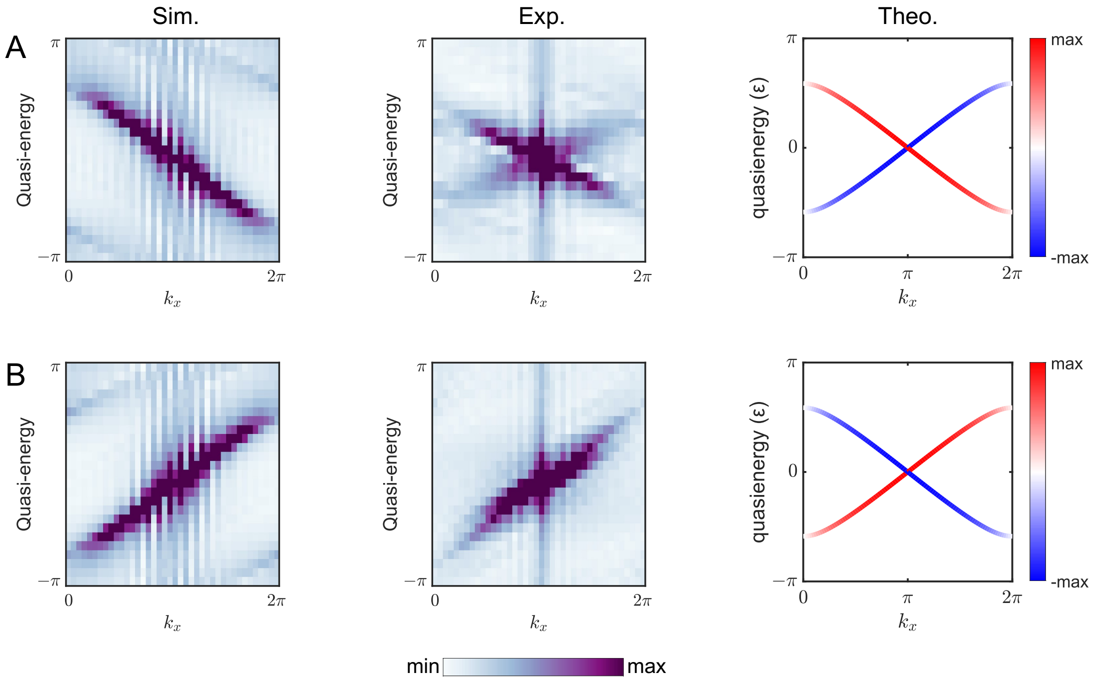

Fig. S8. Determination of the energy band from evolution.

(A) Simulated, experimentally measured, and theoretically calculated energy bands for non-reciprocal coupling in the negative direction. (B) Simulated, experimentally measured, and theoretically calculated energy bands for non-reciprocal coupling in the positive direction.

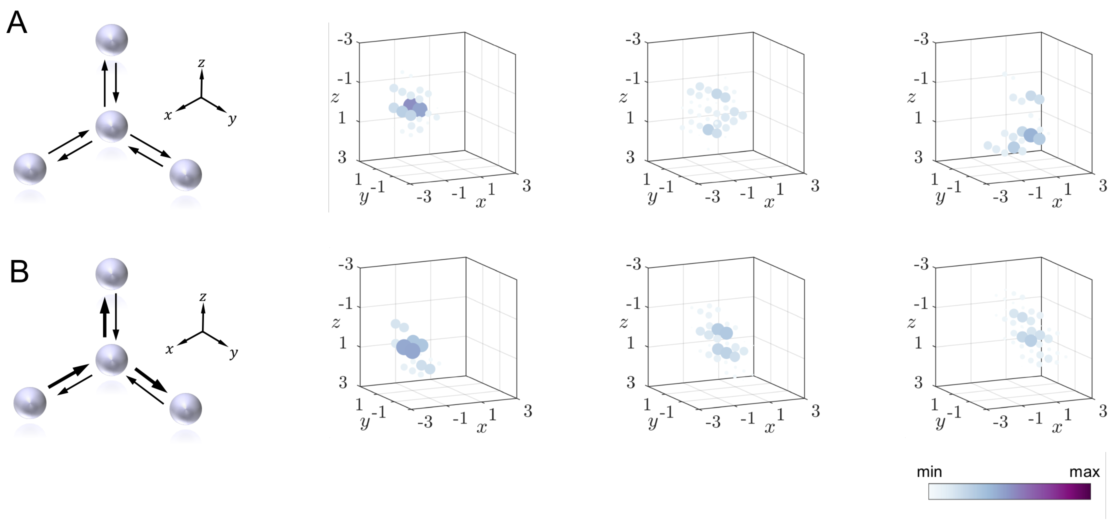

Fig. S9. Extended experiment results in 3D space.

(A) Experiment results for 3D Hermitian evolution for $T = 2, 4$ and 6. (B) Experiment results for 3D Hermitian evolution for $T = 2, 4$ and 6.

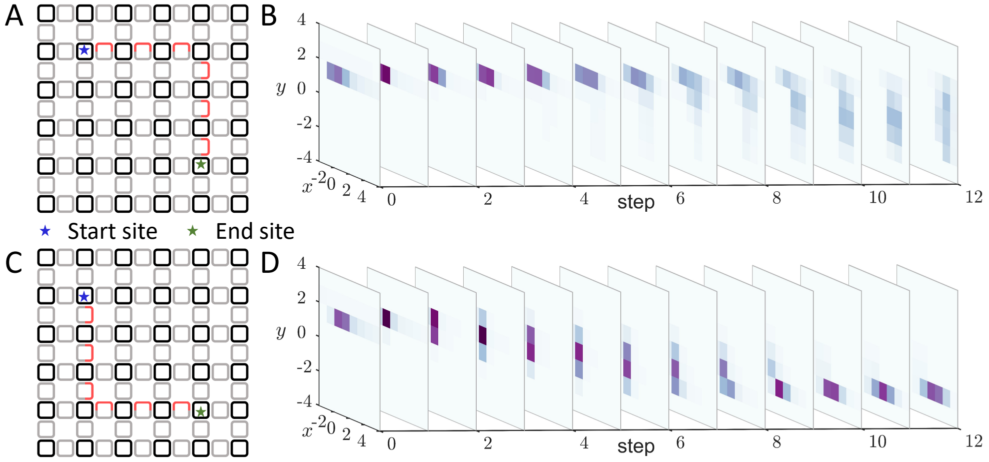

Fig. S10 Results for non-Hermitian router.

(A) The schematic diagram illustrates a non-Hermitian optical router where the red-colored gain region on the link ring facilitates directional light path formation. (B) Experiment results of state evolution along non-Hermitian path. (C) An alternative transmission path configuration. (D) Experiment results of state evolution along non-Hermitian path.

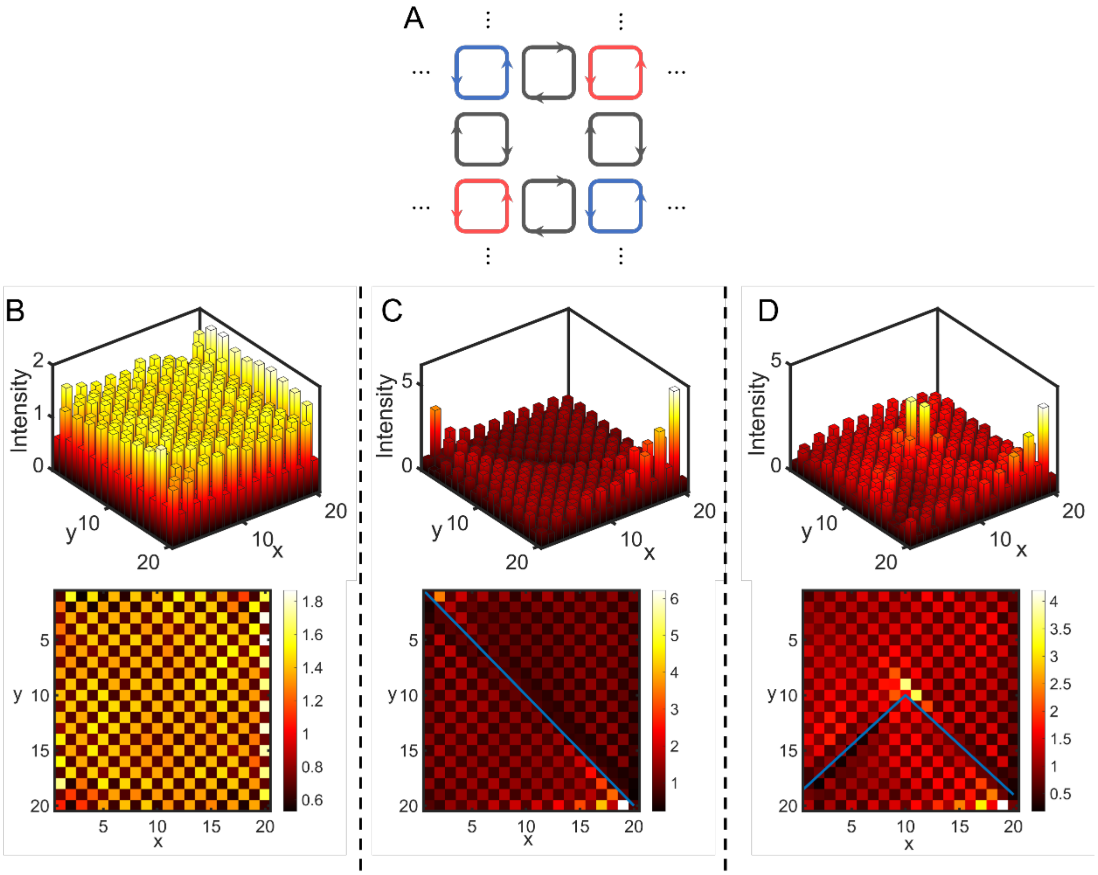

Fig. S11. Realization of the geometry-dependent skin effect.

(A) Schematic diagram of the proposed Hamiltonian. (B) Eigenstate distribution for the square region, where no skin effect is observed. (C) Eigenstate distribution for two right triangular regions, where the skin effect is present. (D) Eigenstate distribution for a triangular region and a concave pentagonal region, where the skin effect is observed in both regions.

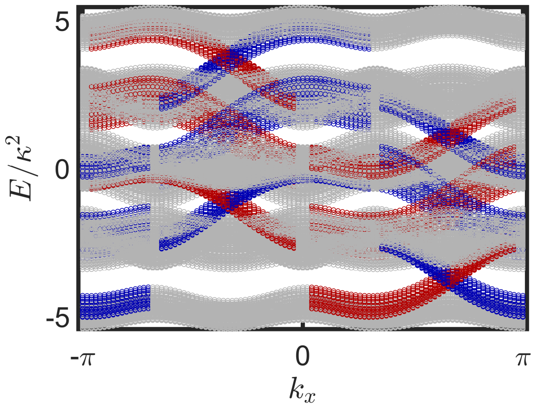

Fig. S12. Energy band of the four-dimensional quantum Hall effect, plotted with k_x as the quantum number.

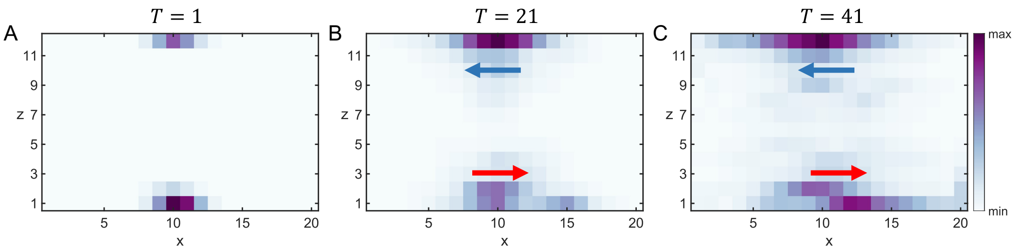

Fig. S13. Chiral transport of edge states in the four-dimensional quantum Hall effect. The intensity distribution of the wave in the $x - z$ projection plane is obtained by summing over y and w after calculating $|\psi|^2$. The evolution steps are (A) 1, (B) 21, (C) 41.
